# Sorting and packaging of RNA into extracellular vesicles shape intracellular transcript levels

**DOI:** 10.1186/s12915-022-01277-4

**Published:** 2022-03-24

**Authors:** Tina O’Grady, Makon-Sébastien Njock, Michelle Lion, Jonathan Bruyr, Emeline Mariavelle, Bartimée Galvan, Amandine Boeckx, Ingrid Struman, Franck Dequiedt

**Affiliations:** 1 Laboratory of Gene Expression and Cancer, GIGA-MBD, University of Liège, B34, Avenue de l’Hôpital 11, 4000 Liège, Belgium; 2 Laboratory of Molecular Angiogenesis, GIGA-Cancer, University of Liège, B34, Avenue de l’Hôpital 11, 4000 Liège, Belgium

**Keywords:** Extracellular vesicles, Exosomes, Gene regulation, mRNA, lncRNA, HNRNPA2B1

## Abstract

**Background:**

Extracellular vesicles (EVs) are released by nearly every cell type and have attracted much attention for their ability to transfer protein and diverse RNA species from donor to recipient cells. Much attention has been given so far to the features of EV short RNAs such as miRNAs. However, while the presence of mRNA and long noncoding RNA (lncRNA) transcripts in EVs has also been reported by multiple different groups, the properties and function of these longer transcripts have been less thoroughly explored than EV miRNA. Additionally, the impact of EV export on the transcriptome of exporting cells has remained almost completely unexamined. Here, we globally investigate mRNA and lncRNA transcripts in endothelial EVs in multiple different conditions.

**Results:**

In basal conditions, long RNA transcripts enriched in EVs have longer than average half-lives and distinctive stability-related sequence and structure characteristics including shorter transcript length, higher exon density, and fewer 3′ UTR A/U-rich elements. EV-enriched long RNA transcripts are also enriched in HNRNPA2B1 binding motifs and are impacted by HNRNPA2B1 depletion, implicating this RNA-binding protein in the sorting of long RNA to EVs. After signaling-dependent modification of the cellular transcriptome, we observed that, unexpectedly, the rate of EV enrichment relative to cells was altered for many mRNA and lncRNA transcripts. This change in EV enrichment was negatively correlated with intracellular abundance, with transcripts whose export to EVs increased showing decreased abundance in cells and vice versa. Correspondingly, after treatment with inhibitors of EV secretion, levels of mRNA and lncRNA transcripts that are normally highly exported to EVs increased in cells, indicating a measurable impact of EV export on the long RNA transcriptome of the exporting cells. Compounds with different mechanisms of inhibition of EV secretion affected the cellular transcriptome differently, suggesting the existence of multiple EV subtypes with different long RNA profiles.

**Conclusions:**

We present evidence for an impact of EV physiology on the characteristics of EV-producing cell transcriptomes. Our work suggests a new paradigm in which the sorting and packaging of transcripts into EVs participate, together with transcription and RNA decay, in controlling RNA homeostasis and shape the cellular long RNA abundance profile.

**Supplementary Information:**

The online version contains supplementary material available at 10.1186/s12915-022-01277-4.

## Background

Extracellular vesicles (EVs) are produced and released by nearly every type of eukaryotic cell and comprise a wide range of membrane-bound extracellular particles. EVs are traditionally classified according to their biogenesis. Exosomes arise from within the endosomal network and are released upon the fusion of a multi-vesicular body with the plasma membrane. Microvesicles (also known as microparticles or ectosomes) are produced by outward budding of the plasma membrane, and apoptotic bodies or blebs are formed by apoptotic cells as they break down [1]. Although this classification scheme suggests that EVs are relatively homogenous entities that can be easily categorized, it is becoming clear that they in fact represent a more heterogeneous collection of subtypes. There are marked differences in vesicles released by different cell types and even the same cell populations can release strikingly different sets of EVs depending on their environmental conditions [2, 3].

Lipids, proteins, and genetic material are all readily detected in EVs, often in proportions that differ dramatically from the contents of their parental cells [4]. Many early reports on EV content focused on proteins [5–7] and new proteomics tools have helped to paint a more global picture of EV peptide content [8]. Similarly, lipidomics are revealing overall EV lipid composition and potential functions [8]. Finally, high-throughput sequencing techniques have provided a wealth of data about the nucleic acid content of EVs, especially RNA [9]. It has been clear for some time that the RNA cargo of EVs skews towards short RNA transcripts [10]. In particular, many reports have focused on miRNAs. EV miRNA contents have been reported for a plethora of cells and the function of EV miRNAs in recipient cells is the subject of intense investigation [11]. Recently, there has been a great interest in miRNA sorting mechanisms, which seem to involve several sequence features and RNA-binding proteins (RBPs) [4]. In addition to miRNA, many other classes of RNA transcripts are present at significant levels in EVs, including piRNAs, tRNAs, snRNAs, mRNAs, and lncRNAs [12–16]. In comparison to miRNAs though, fewer reports have shed light on longer RNA transcripts in EVs at a global level.

At present, EVs are typically viewed as intercellular messengers exported by one tissue or cell type and traveling, sometimes a great distance, to impact recipient cells [1]. In this model, noncoding RNAs are especially important as some have been shown to retain their regulatory function once delivered in the recipient cells [11]. Similarly, mRNA EV cargos can be translated into functional polypeptides in the recipient cells [10]. This remarkable ability to transfer functional molecules from one cell to another has, so far, overshadowed questions about the relationship of EVs to their cells of origin. Here, we present evidence that the packaging of long RNA molecules into EVs impacts the abundance of those transcripts within the parent cells. These results raise the exciting possibility that EV export participates in shaping the transcriptome of the producing cell.

## Results

### Extracellular vesicles contain long and short RNA transcripts

**Fig. 1:**
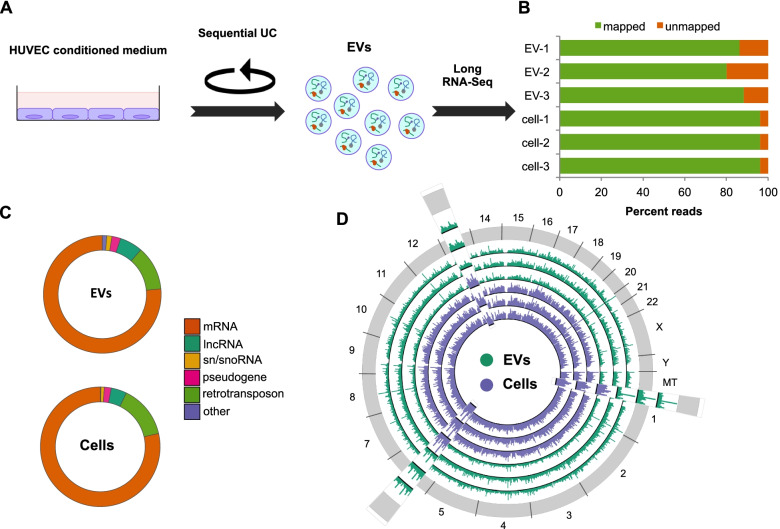
Long RNA content of EVs. **A** Experimental overview. **B** Percentage of RNA-Seq reads mapped to the human genome or unmapped from 3 EV and 3 cell samples. Individual values can be found in Additional file 17. **C** RNA-Seq reads from 3 replicates mapped to known RNA transcripts by class of RNA transcript. rRNA reads are excluded. Individual values can be found in Additional file 17. **D** Circos plot of RNA-Seq reads from 3 EV and 3 cell samples mapped to the human genome. Three regions are expanded to more clearly show differences

EVs were obtained from HUVEC cultures using a standard ultracentrifugation protocol (Fig. 1A). We confirmed that our preparations were enriched in several EV markers and depleted of cellular markers relative to cells [17] (Additional file 1: Fig. S1A). On average, EVs in our preparations exhibited sizes of around 100–120 nm (Additional file 1: Fig. S1B). Cryo-electron microscopy revealed heterogeneity in EV morphology. Most of the vesicles were intact, isolated, and round-shaped and had a single clear lipid bilayer membrane (Additional file 1: Fig. S1C). Many of the EVs with a size around 100 nm appeared filled with electron dense cargo. In contrast, larger vesicles often had lighter inside material. Altogether, these observations illustrate the heterogeneity of the EV population produced by ultracentrifugation from HUVEC supernatant.

For this project, we focused on long RNA transcripts as their presence and condition in EVs have been less characterized compared to short RNAs. Most second-generation RNA-Seq library preparation methods rely on transcript fragmentation early in the protocol, leading to ambiguity as to whether input long RNA transcripts were intact or already fragmented, which remains a controversial issue for EVs [10, 18–23]. To optimize recovery of long RNAs, we used the Nugen Ovation SoLo RNA-Seq system. Like those observed from other cell types [10, 12, 19, 21, 24, 25], our EV preparations were enriched for short RNA. However, using this library preparation method, we were able to selectively capture the longer RNA molecules that were present (Additional file 1: Fig. S1D). Illumina sequencing of the RNA libraries yielded good quality reads, with a high proportion of reads mapping to the human genome for both cells and EVs (Fig. 1B).

RNA-Seq analysis revealed a large diversity of long RNA transcripts in EVs. Overall, the proportions of different RNA species detected were comparable in cells and EVs, with the majority of the reads mapping to mRNA (Fig. 1C). Interestingly, peaks in read coverage frequently occurred in different genomic locations for cells and for EVs (Fig. 1D), suggesting that the repertoire of long RNA transcripts is different between cells and EVs, as has been well established for short RNA transcripts [4].

### Long RNA transcripts are unevenly distributed between cells and EVs in functionally relevant patterns

**Fig. 2:**
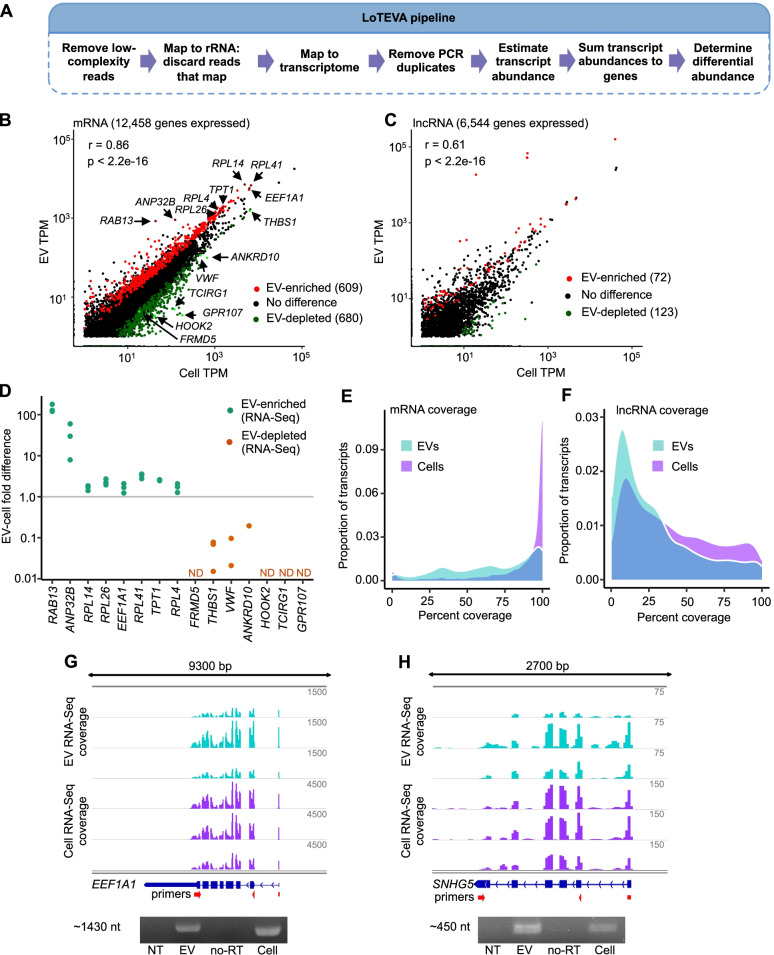
Long RNA transcripts in EVs are full-length and differ from cells**. A** Overview of LoTEVA pipeline for EV RNA-Seq analysis. **B** mRNA abundance from RNA-Seq in EVs and cells. Genes indicated by name were validated by qRT-PCR. TPM = transcripts per million. TPM values are averaged across 3 replicates. **C** lncRNA abundance from RNA-Seq in EVs and cells. TPM = transcripts per million. TPM values are averaged across 3 replicates. **D** Fold difference determined by qRT-PCR from 3 independent samples for select EV-enriched and EV-depleted transcripts. Individual values can be found in Additional file 17. **E** RNA-Seq read coverage of mRNA transcripts in EVs and cells. *N* = 3. **F** RNA-Seq read coverage of lncRNA transcripts in EVs and cells. *N* = 3. **G** RNA-Seq read coverage (top) and RT-PCR amplicons (bottom) of *EEF1A1* mRNA. NT = no template, no-RT = RNA without reverse transcriptase. **H** RNA-Seq read coverage (top) and RT-PCR amplicons (bottom) of lncRNA *SNHG5*. NT = no template, no-RT = RNA without reverse transcriptase. Uncropped images of gels can be found in Additional file 18

To further investigate the idea of selective packaging of long RNA transcripts, we developed a Long Transcript EV Abundance (LoTEVA) analysis method: a robust, multi-step informatics pipeline that addresses EV-specific sequencing issues to estimate abundance levels and test for differential abundance of specific long RNA transcripts between cells and EVs (Fig. 2A). In the first step of the LoTEVA pipeline, low-complexity and rRNA reads are removed from the analysis. Next, reads are mapped to the human transcriptome with Salmon software. Salmon allows mapping and estimated counting of reads that map to multiple different transcripts. We chose this method, instead of the alternative of working only with uniquely mapping reads, because some bona fide RNA transcripts can arise from multiple locations in the genome (e.g., snRNAs) or contain regions of similarity to other genes [26], and because a higher proportion of reads from EVs than from cells consistently maps to multiple locations in the genome (ref. [19] and Additional file 2: Fig. S2A). Next, because PCR duplication is a concern with low-input sequencing as is typical with EV genetic material [27], LoTEVA identifies reads that have matching molecular barcodes from library construction and map within a half-read length of each other on the transcriptome, discarding all matching reads but one. Finally, the high-quality remaining reads are re-mapped with Salmon to estimate transcript abundance and the DESeq2 method is used to determine whether abundance levels of transcripts are significantly different between cells and EVs.

After estimating transcript abundance in our cell and EV samples, we used hierarchical clustering and observed that EV RNA samples clustered tightly together and apart from cellular RNA samples (Additional file 2: Fig. S2B). The LoTEVA pipeline reliably detected 12,458 protein-coding and 6544 lncRNA genes expressed in cells, of which 10,761 mRNA and 2393 lncRNA were also detected in EVs. Because others have noted over- and underrepresentation of different RNA classes in EVs [12, 19, 20], we evaluated differential abundance for mRNA and lncRNA separately from other classes of RNA. Overall, abundance of transcripts in EVs correlated linearly with abundance of transcripts in cells (*r* = 0.86, *p* < 2.2e−16 for mRNA; *r* = 0.61, *p* < 2.2e−16 for long noncoding RNA), indicating that long RNA EV content is largely a reflection of the cellular transcriptome (Fig. 2B, C). However, differential abundance analysis revealed that transcripts of 609 protein-coding genes and 72 lncRNA genes were enriched in EVs relative to cells, while transcripts of 680 protein-coding genes and 123 lncRNA genes were depleted in EVs relative to cells (Additional file 3). These differences in EV and cellular abundance were validated by qRT-PCR analysis of selected RNAs, including both enriched and depleted transcripts (Fig. 2D).

Whether long RNA transcripts are present in EVs as full-length molecules or fragments is difficult to assess globally by common RNA sequencing methods and remains a controversial issue. Evidence for the presence of full-length transcripts [10, 21], fragmented transcripts [22, 23], and both [18–20] has been reported. Our pipeline uses an RNA-Seq library preparation method that was specifically formulated to select for longer RNA molecules; however, this alone does not establish that full-length mRNA and lncRNA transcripts are indeed present in EVs. To more thoroughly examine the state of long RNA in EVs, we began by evaluating the extent of RNA-Seq read coverage across individual transcripts. For each gene to which RNA-Seq reads mapped, we used the most 5′ and most 3′ mapping reads within the coding region (in the case of protein-coding genes) or full transcript (in the case of lncRNA) to estimate coverage, then compared transcriptome-wide distributions of read coverage for RNA obtained from cells and from EVs (Fig. 2E, F). Using the proportion of the coding sequence (for mRNAs) or transcript length (for lncRNAs) covered by the sequence reads, we found that protein-coding transcripts typically showed a greater extent of coverage (average 85.0% in cells and 65.9% in EVs) than lncRNA transcripts (average 40.1% in cells and 28.9% in EVs), possibly reflecting less complete annotation of lncRNAs than mRNAs. Cells had a high proportion of transcripts with nearly 100% read coverage, indicating that most of the cellular long transcriptome is made up of full-length molecules, as expected. However, cells also had many transcripts for which full-length coverage was not apparent; for these, it is possible that (i) the transcripts are present as fragments, (ii) the standard annotation does not match the biological reality in this cell type, or (iii) the depth of RNA sequencing was not sufficient to obtain read coverage along the length of less abundant transcripts. While EV RNA showed more variation in coverage, a significant proportion of EV-associated transcripts showed end to end read coverage, indicating that they might be full-length transcripts.

To directly test this possibility, we investigated the integrity of selected transcripts using RT-PCR, including transcripts of 5 protein-coding genes (*EEF1A1*, *HNRNPA1*, *ANP32B*, *RPL14*, and *RPL41*) and 2 lncRNA genes (*SNHG5* and *GAS5*). We opted to test for the presence of annotated coding sequences (for mRNA) or 90% of annotated sequence (for lncRNA) rather than testing for the entire annotated sequence in order to reflect that untranslated region (UTR) lengths can vary in cells relative to standard annotation (ref. [28], and see the shorter 3′ UTR in RNA-Seq coverage in Fig. 2G). For each tested transcript and for both cells and EVs, we amplified a fragment of the expected size, ranging from 100 nt for *RPL41* up to 1430 nt for *EEF1A1* (Fig. 2G, H, Additional file 2: Fig. S2D-H). Interestingly, patterns of read coverage across transcripts were not notably different between cells and EVs, with high coverage of exons and scant coverage of introns. Altogether, these results indicate that long, continuous mature transcripts of both mRNA and lncRNA are present in EVs (see Fig. 2G, H and Additional file 2: S2D-H for examples). However, we cannot exclude that some long RNAs found in EVs might be fragmented, or present as a mixture of full-length and fragmented transcripts.

### Long RNA transcripts enriched in EVs show characteristic differences

**Fig. 3:**
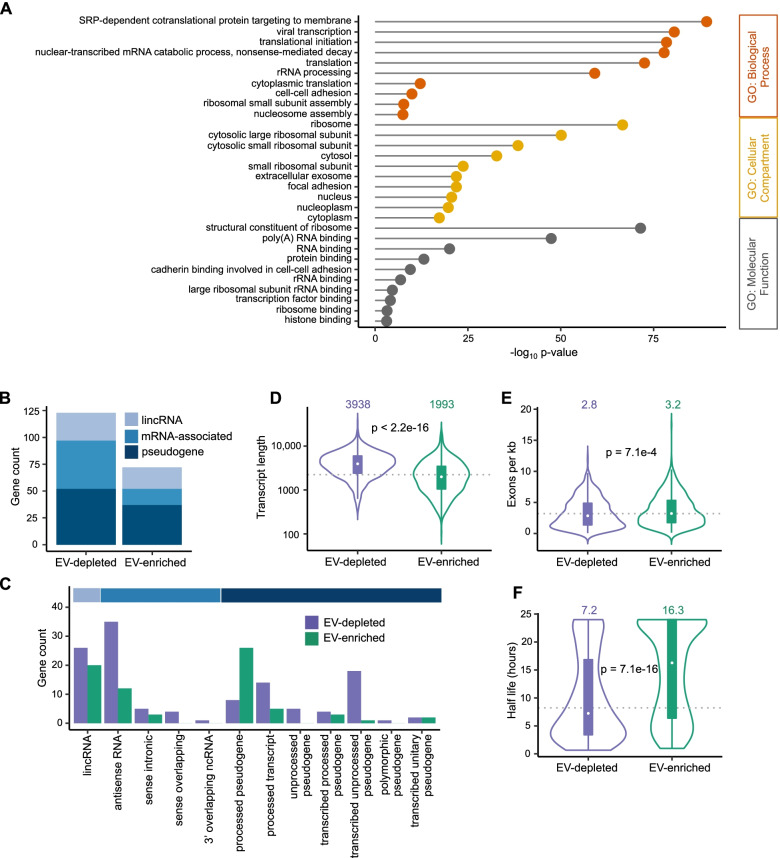
EV-enriched and EV-depleted transcripts have different characteristics. **A** Gene Ontology analysis of EV-enriched protein-coding genes performed with DAVID. For each GO category the ten significant (FDR < 0.05) terms with the lowest *p*-values are displayed. Individual values can be found in Additional file 17. **B** Broad categories of long noncoding RNAs enriched or depleted in EVs. Gray = long intergenic noncoding RNA, light blue = mRNA-associated lncRNA (antisense, intronic, or overlapping), navy = pseudogene. Individual values can be found in Additional file 17. **C** Specific gene biotypes of long noncoding RNAs enriched or depleted in EVs. Individual values can be found in Additional file 17. **D** Violin plot of transcript length for EV-enriched and EV-depleted genes. **E** Violin plot of number of exons per kilobase of transcript in EV-enriched and EV-depleted genes. **F** Violin plot of transcript half-life in actinomycin-D-treated HeLa cells for EV-enriched and EV-depleted transcripts. For all violin plots, medians are indicated above each violin and grey dotted lines indicate median of all expressed genes. *P*-values calculated by Welch two-sample *t*-test are indicated. All analyses were performed using 3 EV and 3 cell samples

A Gene Ontology (GO) analysis revealed that the EV-enriched mRNA transcripts were statistically enriched for several GO terms, supporting the idea that sorting and loading of transcripts into EVs are non-random. Transcripts preferentially exported into EVs were enriched for GO terms related to RNA transcription and translation, such as *SRP-dependent cotranslational protein targeting to membrane* (GO:0006614), *translational initiation* (GO:0006413), and *cytoplasmic translation* (GO:0002181) (Fig. 3A). No specific GO term was significantly enriched when analyzing mRNA transcripts that were preferentially retained in cells, i.e., depleted in EVs.

Next, we examined the lncRNAs that were preferentially exported to EVs or retained in cells. Long intergenic noncoding RNAs (lincRNAs), mRNA-associated long noncoding RNAs (antisense RNAs, sense intronic RNAs, sense overlapping RNAs and 3′ overlapping ncRNAs) and pseudogenes all showed some level of differential distribution, with some transcripts being preferentially exported to EVs and others being preferentially retained in cells (Fig. 3B, C, Additional file 3). Because regulatory relationships have been described between antisense lncRNAs and protein-coding (PC) genes, we paired the antisense (AS) lncRNAs expressed in cells with the PC genes they lie opposite to [29], and compared EV enrichment/depletion of the pairs (Additional file 4: Fig. S3A). We did not observe a positive or negative correlation of EV packaging between the pairs. Similarly, at the transcriptional level, lincRNAs have been reported to be frequently coregulated with the protein-coding genes located nearest to them on the genome [30]. We paired the lincRNAs expressed in cells with their nearest-neighbor protein-coding genes and compared EV packaging between the pairs. We did not observe any positive or negative correlation of EV export between the pairs (Additional file 4: Fig. S3B). These data suggest that, while lncRNA packaging into EVs is regulated at the transcript level, this packaging does not appear to be controlled by lncRNA subtype or by the mechanisms governing transcriptional relationships between some lncRNAs and their associated protein-coding RNAs.

We next investigated whether long RNAs preferentially exported into EVs displayed any specific features as compared to those retained in cells. EV-enriched transcripts were significantly shorter than EV-depleted transcripts (median 1993 nt vs. 3938 nt, *p* < 2.2e−16, Welch’s *t*-test, Fig. 3D). The length difference was apparent for both mRNAs and lncRNAs, and correlated with the observation that EV-enriched mRNA transcripts had shorter coding sequences (CDS) (median 783 nt for EV-enriched vs. 1905 nt for EV-depleted, *p* < 2.2e−16, Welch’s *t*-test) and 3′ UTRs (median 708 vs. 1496 nt, *p* = 2.9e−11, Welch’s *t*-test), while the length of their 5′ UTRs was not significantly different (median 176 vs. 198 nt, *p* = 0.374, Welch’s *t*-test, Additional file 4: Fig. S3C-G). Interestingly, although they were shorter on average, EV-enriched mRNA and lncRNA transcripts had more exons relative to their length than did cell-enriched transcripts (3.2 vs. 2.8 exons per kilobase, *p* = 4.0e−3, Welch’s *t*-test, Fig. 3E, Additional file 4: Fig. S3H-I). Features associated with EV-enriched transcripts, e.g., shorter 3′ UTR and transcript length and increased exon density, have been linked with increased RNA stability [31–33]. To investigate the stability of EV-enriched transcripts, we obtained transcriptome-wide half-life data from both immortalized primary (LCL) cells [34, 35] and cancer cells (ref. [36, 37] and our lab). Even though these studies were performed in different cell lines and used different methodologies to estimate cellular RNA half-lives, we consistently found that EV-enriched transcripts had significantly longer half-lives than EV-depleted transcripts (Fig. 3F, Additional file 4: Fig. S3J-L). In addition, 3′ UTRs but not 5′ UTRs of EV-enriched mRNA contained on average a significantly lower density of AU-rich elements (AREs), which are cis-acting elements that promote mRNA instability [33] (Additional file 4: Fig. S3M-N). Altogether, our results indicate that EV-enriched mRNA and lncRNA transcripts display features that are commonly associated with stable RNA, including high exon density, shorter length, shorter 3′ UTRs, and lower frequency of 3′ UTR AREs.

**Fig. 4:**
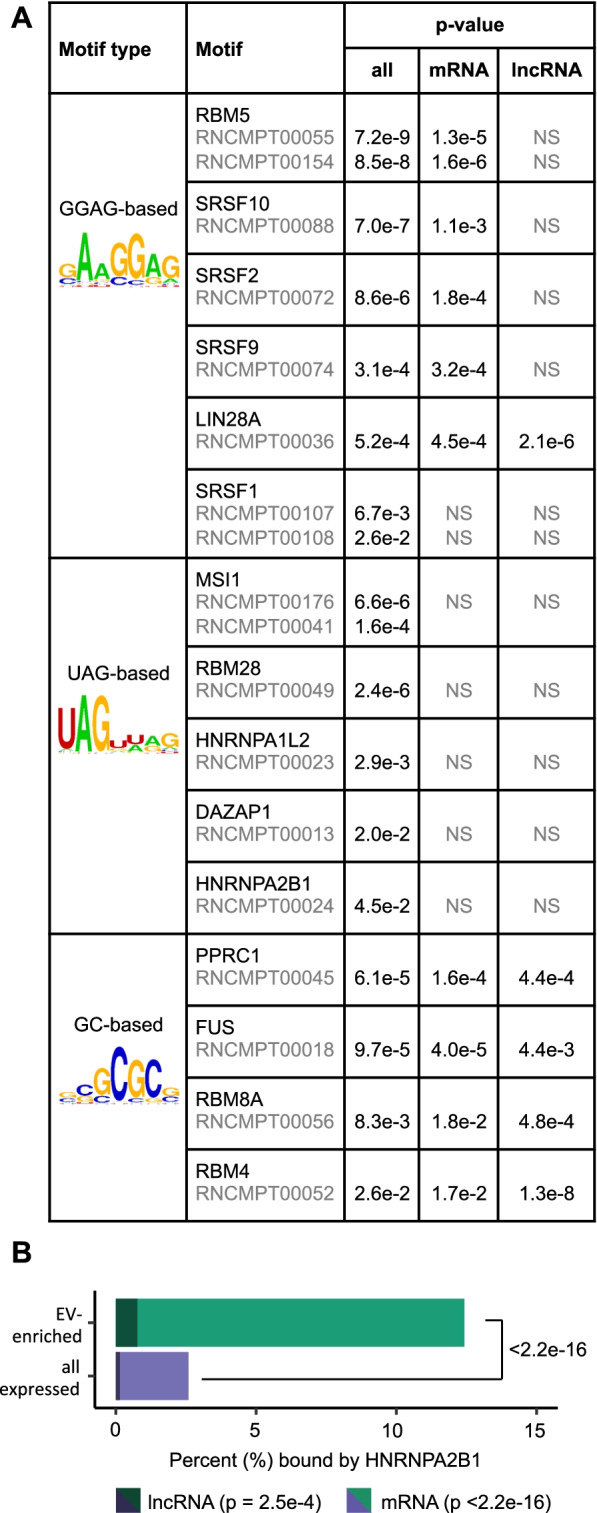
EV-enriched transcripts and RBPs. **A** Enriched motifs from AME analysis in EV-enriched mRNA and lncRNA transcripts (padj < 0.1) relative to unchanged mRNA and lncRNA transcripts in 3 EV samples relative to 3 cell samples. RBP name, *p*-value and motif ID are shown. **B** Number of expressed and EV-enriched mRNA and lncRNA genes bound by HNRNPA2B1, as determined by HITS-CLIP. *P*-value determined by Fisher’s exact test. *N* = 16,249 expressed genes (11,825 mRNA and 4424 lncRNA), 652 EV-enriched genes (561 mRNA and 91 lncRNA). Individual values can be found in Additional file 17

To identify additional sequence features of EV-enriched long RNA, we performed an Analysis of Motif Enrichment (AME) using the MEME suite [38]. We found that long transcripts preferentially exported to EVs were enriched for several RNA motifs associated with specific RNA-binding proteins. While we observed significant and consistent motif enrichment by examining either mRNA or lncRNA separately, combining these two subgroups afforded us more statistical power and allowed detection of more enriched motifs. The AME analysis identified GC-rich motifs bound by proteins such as PPRC1, RBM4, FUS, and RBM8A (Fig. 4A). This is consistent with the observation that EV-enriched mRNA and lncRNA had a higher percentage of G/C nucleotides than did cell-retained transcripts (50.6% vs. 48.3%, *p* = 8.6e−3, Welch’s *t*-test, Additional file 5: Fig. S4A-C). We also observed significant enrichment in UAG-containing motifs, which are bound by proteins including HNRNPA1, HNRNPA2B1, and RBM28, as well as enrichment in GGAG-containing motifs, which are typically bound by proteins including RBM5 and LIN28A (Fig. 4A). These GGAG-containing motifs bear a high similarity to an EXOmotif found in EV-enriched miRNAs by Villarroya-Beltri et al. [39]. This EXOmotif is bound by HNRNPA2B1 and plays a major role in the sorting of some miRNAs into EVs by primary T lymphoblasts. HNRNPA2B1 is also known to regulate the packaging of miRNA into endothelial cell EVs, although apparently without the involvement of the GGAG motif [40], and has been shown to be involved in the targeting of specific lncRNAs into cancer cell EVs [41, 42]. Based on these findings, we hypothesized that HNRNPA2B1 might be involved in sorting long transcripts out of endothelial cells into EVs. To examine whether the EV-enriched transcripts are indeed bound by HNRNPA2B1, we obtained HNRNPA2B1-RNA binding site data from a HITS-CLIP experiment [43, 44]. We found that EV-enriched mRNA and lncRNA transcripts were nearly 5 times as likely as expressed mRNA and lncRNA in general to contain HNRNPA2B1 binding sites (Fig. 4B, 12.4% of EV-enriched transcripts vs. 2.6% of EV-depleted transcripts, *p* < 2.2e−16).

### Transcript packaging into EVs is inversely correlated with cellular transcript abundance changes

**Fig. 5:**
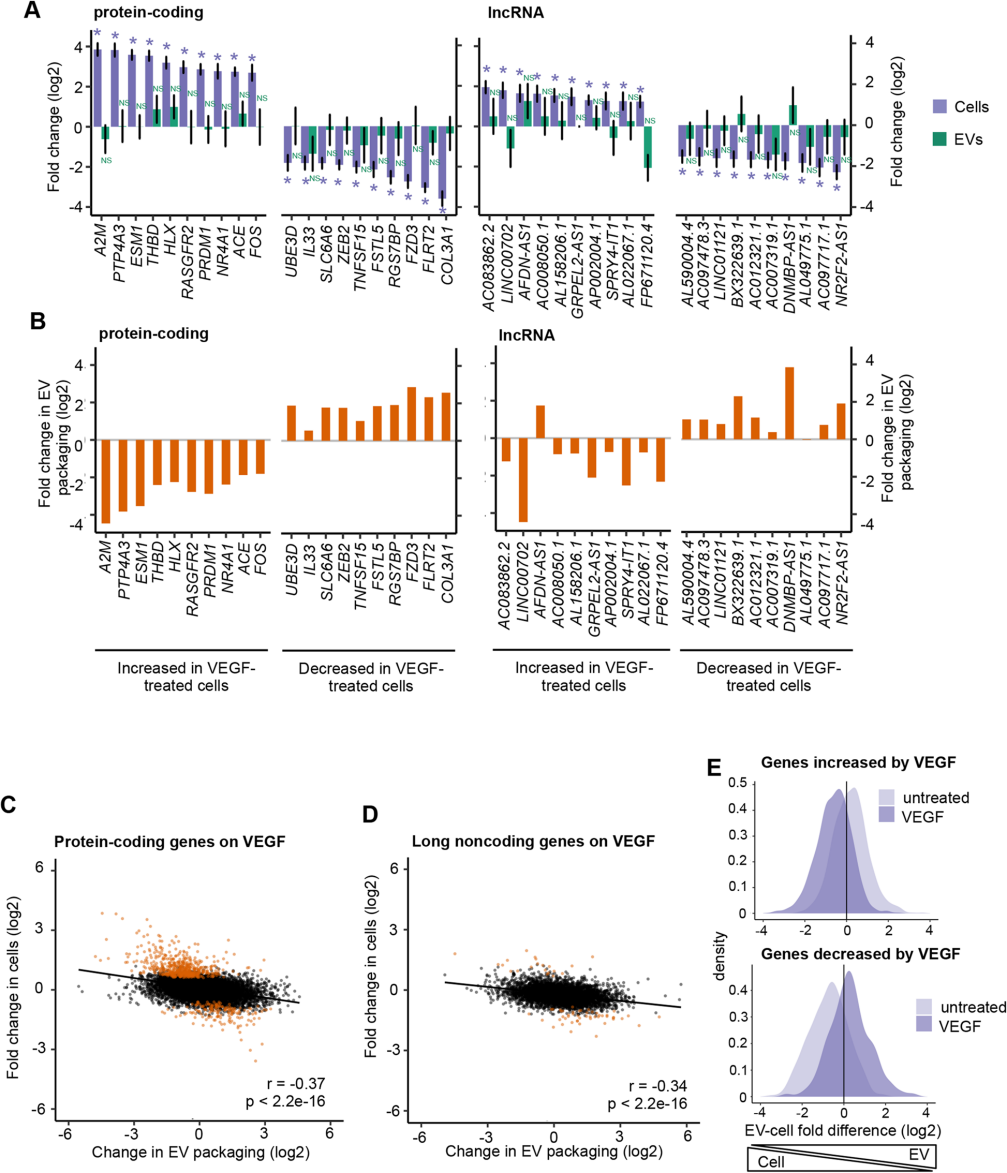
Changes in transcript packaging are negatively correlated with changes in cellular abundance. **A** Log2 fold change in VEGF-treated cells vs. untreated cells and in EVs derived from VEGF-treated cells vs. EVs derived from untreated cells for the most increased and decreased protein-coding and lncRNA genes significantly changed in cells. Error bars represent standard error of log2 fold change. * = adjusted *p*-value < 0.1, NS = adjusted *p*-value > 0.1. Individual values can be found in Additional file 17. **B** Log2 change in packaging for the most increased and decreased protein-coding and lncRNA genes in cells. Individual values can be found in Additional file 17. **C** Log2 change in EV packaging and log2 fold change in cell abundance for protein-coding genes upon VEGF treatment. Orange = adjusted *p*-value < 0.1 in VEGF-treated vs. untreated cells. **D** Same as **C** for long noncoding genes. **E** Distribution of log2 fold differences of protein-coding and lncRNA genes (combined) between EVs and cells in genes increased in cells by VEGF treatment (top) or decreased in cells by VEGF treatment (bottom). Light purple = log2 fold difference distribution in untreated cells. Dark purple = log2 fold difference distribution in VEGF-treated cells. All analyses were performed using 3 EV and 3 cell samples

EV contents are sometimes regarded as “snapshots” of parental cell content, with cargos that can be used as biomarkers reflecting the condition of parental cells, in particular in states of disease or stress. Indeed, we observed a strong positive correlation between EV and cellular abundances for most long RNA transcripts (Fig. 2B, C). To investigate to what extent EV long RNA profiles reflect those of their parent cells in changing conditions, we altered the HUVEC transcriptome with recombinant vascular endothelial growth factor (VEGF)_165_, a well-described pro-angiogenic factor [45]. We then collected EVs from VEGF-stimulated cells and harvested RNA in parallel from EVs and cells. As observed for untreated cells (Fig. 2B, C), mRNA and lncRNA abundances in EVs from VEGF-activated cells still largely reflected abundances in cells (*r* = 0.942, *p* < 2.2e−16 for mRNA; *r* = 0.828, *p* < 2.2e−16 for lncRNA) (Additional file 6: Fig. S5A-B, Additional file 7). In HUVEC, VEGF treatment led to the expected upregulation of a high number of genes associated with angiogenic activation, including genes involved in cell proliferation, permeability, and adhesion (Additional file 6: Fig. S5C, Additional file 8). VEGF treatment also impacted the repertoire of long transcripts exported into EVs, but to a much lesser extent than the cellular transcriptome (Additional file 6: Fig. S5D-E, Additional file 9). Surprisingly, very few of the transcripts whose abundance levels changed in cells showed corresponding changes in EVs. In particular, of the 10 mRNA and lncRNA genes showing the largest increases or decreases in transcript abundance in cells, none were significantly affected in EVs (Fig. 5A). This lack of correspondence between changes in cells and changes in EVs called into question the assumption that EV contents faithfully represent cell contents. Interestingly, when we investigated EV packaging of these highly changed genes after VEGF treatment, in nearly all cases we saw a decrease in EV-enrichment relative to cells for the genes that increased in cells, and an increase in EV-enrichment relative to cells for the genes that decreased in cells (Fig. 5B). This observation raised the surprising possibility that cells modulate the extent of EV packaging of specific transcripts in changing conditions. To globally investigate this issue, we compared the changes in EV-packaging of mRNAs and lncRNAs to changes in intracellular levels between the VEGF-activated and non-activated conditions. Interestingly, across the transcriptome, the ratio of EV packaging in VEGF *vs*. untreated conditions showed a small but significant negative correlation with the changes in intracellular transcript abundances (Fig. 5C–E). Both mRNA (Fig. 5C) and lncRNA (Fig. 5D) transcripts that decreased in cells upon VEGF treatment tended to be more heavily packaged into EVs, while in contrast, transcripts that increased in cells in response to VEGF were in general less packaged into EVs. This observation raised the exciting possibility that packaging of mRNA and lncRNA transcripts into EVs plays a role in the regulation of intracellular transcript abundance.

**Fig. 6:**
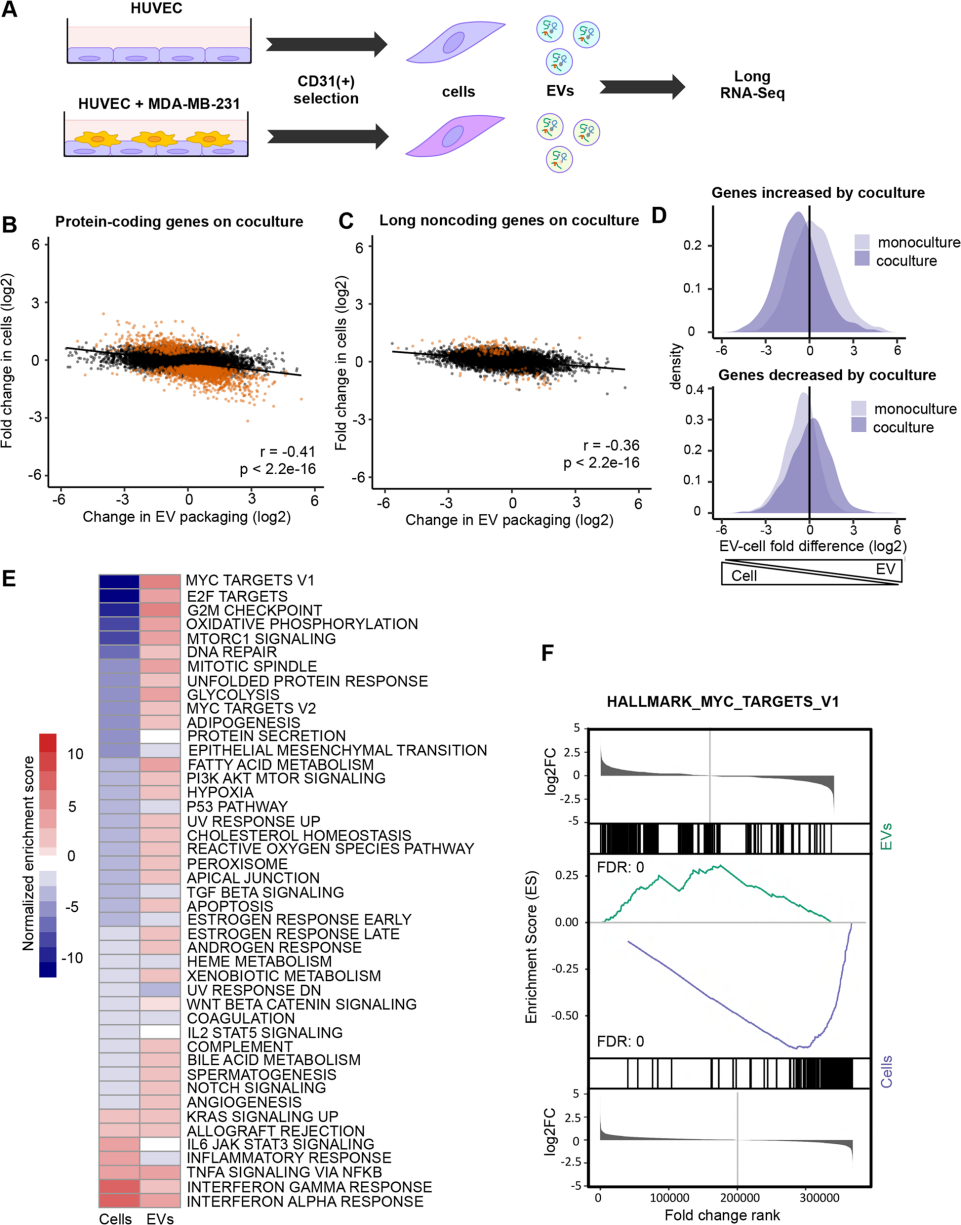
Changes in transcript packaging are negatively correlated with changes in cellular abundance. **A** Overview of tumor cell exposure procedure. **B** Log2 change in EV packaging and log2 fold change in cell abundance for protein-coding genes upon coculture. Orange = adjusted *p*-value < 0.1 in cocultured vs. monocultured cells. **C** Same as **B** for long noncoding genes. **D** Distribution of log2 fold differences of protein-coding and lncRNA genes (combined) between EVs and cells in genes increased in cells by coculture (top) or decreased in cells by coculture (bottom). Light purple = log2 fold difference distribution in monoculture cells. Dark purple = log2 fold difference distribution in cocultured cells. **E** GSEA analysis of gene sets enriched or depleted in cocultured vs. monocultured cells (left column) and in EVs derived from cocultured vs. monocultured cells (right column). All gene sets shown have FDR < 0.1 in cells. Individual values can be found in Additional file 17. **F** Depletion of MYC_TARGETS_V1 gene set in cocultured vs. monocultured cells and enrichment of the same gene set in EVs derived from cocultured vs. monocultured cells. All analyses were performed using 3 EV and 4 cell samples

It has been extensively documented that endothelial cells modify their gene expression program when exposed to tumor cells (e.g., refs [46, 47].). To extend our observations to more physiological conditions, we cocultured HUVEC with metastatic breast cancer MDA-MB-231 cells (Fig. 6A). We then used magnetic beads to separate both the endothelial cells and their corresponding EVs from tumor cells and tumor-derived EVs, extracted RNA from the cells and EVs, and analyzed cell and EV RNA contents with the LoTEVA pipeline (Additional files 10, 11, 12, 13). Genes whose expression was modified in tumor-exposed endothelial cells were, as expected, enriched for GO terms related to migration and proliferation, such as *cell-cell adhesion* (GO:0098609) and *cell division* (GO:0051301), among others (Additional file 14: Fig. S6A). As observed following VEGF stimulation, while transcript levels changed in EVs (Additional file 14: Fig. S6B), they did not recapitulate the changes observed in cells (Additional file 14: Fig. S6C). When we compared coculture-induced changes in EV-packaging of mRNA and long lncRNA transcripts to the corresponding changes in the parental cells, we again observed the negative correlation that we observed in the VEGF experiment (Fig. 6B–D). Strikingly, this anticorrelation extended to the pathway level for mRNA: gene sets and pathways that *decreased* in the endothelial cells cocultured with tumor cells were in most cases *increased* in the corresponding EV mRNA set (Fig. 6E, F, Additional file 14: Fig. S6D-E), supporting the idea that export of long RNA transcripts into EVs impacts the parent cell transcriptome in functionally consequential ways.

### Inhibition of EV biogenesis alters the cellular transcriptome

**Fig. 7:**
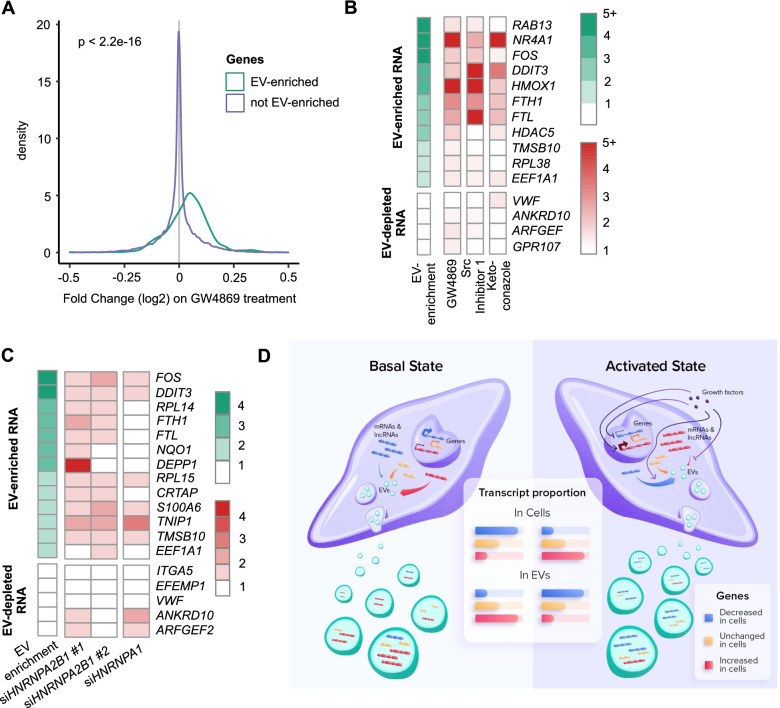
Inhibiting EV secretion or RNA packaging affects cellular transcript levels. **A** Distributions of RNA-Seq log2 fold changes of genes enriched in EVs (green) and genes not enriched in EVs (purple) in cells treated with GW4869. *N* = 3. **B** Enrichment in EVs vs. cells by RNA-Seq (green scale; *n* = 3) and fold changes by qRT-PCR of selected genes in cells after treatment with GW4869, Src Inhibitor 1, or Ketoconazole compared to control cells treated with DMSO. Results are means from *n*=9, 4, and 5 independent experiments for GW4869, Src Inhibitor 1, and Ketoconazole, respectively. Individual values can be found in Additional file 17. **C** Enrichment in EVs vs. cells by RNA-Seq (green scale; *n* = 3) and fold changes by qRT-PCR of selected genes after treatment with two siRNA against *HNRNPA2B1* or *HNRNPA1* compared to cells treated with control siRNA. Results are means from 6, 5, and 4 experiments for si*HNRNPA2B1*#1, si*HNRNPA2B1*#2, and si*HNRNPA1*, respectively. Individual values can be found in Additional file 17. **D** Model for regulation of intracellular mRNA and lncRNA levels by packaging into EV: increased packaging of mRNA and lncRNA RNA transcripts into EVs leads to decreased abundance of those transcripts in the cell. Decreased packaging of mRNA and lncRNA transcripts into EVs leads to increased abundance of those transcripts in the cell

The above observations led us to raise the exciting hypothesis that cells might package specific mRNA and lncRNA transcripts to control their intracellular levels. To test this, we treated HUVEC cultures with the exosome inhibitor GW4869 [48] and quantified cellular changes in long RNA levels by RNA-Seq (Additional file 15: Fig. S7A, Additional file 16). The average cellular abundance of transcripts that are EV-enriched in basal conditions was significantly increased in cells upon GW4869 treatment (*p* < 2.2 e−16 by Welch two-sample *t*-test, Fig. 7A), an observation that held true for both mRNA (*p* < 2.2 e−16, Additional file 15: Fig. S7B) and lncRNA (*p* = 5 e−4, Additional file 15: Fig. S7C). In contrast, GW4869 had no consistent effect on mRNA and lncRNA transcripts that are not normally enriched in EVs relative to cells, suggesting that blocking EV biogenesis specifically increases intracellular levels of transcripts that are normally exported to EVs. To validate these observations with other inhibitors of EV biogenesis, we also tested the effects on the cellular transcriptome of a specific Src inhibitor (Src inhibitor 1) and ketoconazole. Both compounds inhibit EV biogenesis (Additional file 15: Fig. S7D-E) but appear to do so through different pathways than GW4869 [49, 50]. Using qRT-PCR, we tested the abundance of a series of representative mRNA transcripts that are normally enriched in EVs relative to cells. In agreement with our model, we found that the cellular abundance of most of these transcripts increased on treatment with EV inhibitors, while mRNA transcripts that are normally depleted in EVs relative to cells were affected little or not at all (Fig. 7B). Interestingly, the extent to which intracellular levels of specific RNAs were affected was different for each EV inhibitor. For instance, *FOS* mRNA levels were increased by GW4869 and Src inhibitor 1, but appeared unaffected by ketoconazole. These observations may reflect that, although these compounds all inhibit release of EVs (Additional file 15: Fig. S7A,D-E and refs [48–50].), they may affect different subsets of EVs, as indicated by the varying levels of cellular transcript increase upon treatment.

Our results above on long coding and noncoding RNA and observations from others on miRNA suggest that HNRNPA2B1 might be implicated in sorting RNA transcripts to EVs. Therefore, we reasoned that preventing HNRNPA2B1-dependent export of transcripts to EVs would lead to an increase in the cellular level of HNRNPA2B1 targets. We identified several EV-enriched mRNA transcripts that had potential HNRNPA2B1-binding sequence motifs and/or significant HNRNPA2B1 binding as determined by HITS-CLIP [43, 44] (Additional file 15: Fig. S7F). We then knocked down HNRNPA2B1 in HUVECs using two different siRNA and evaluated the cellular levels of these target transcripts. As expected, the cellular abundance of most of these EV-enriched HNRNPA2B1 target transcripts was increased upon knockdown of HNRNPA2B1. Importantly, levels of control EV-depleted mRNA transcripts remained unaffected. In addition, knockdown of the closely related HNRNPA1 did not recapitulate these observations, leading to the upregulation of fewer EV-enriched and more EV-depleted mRNA (Fig. 7C, Additional file 15: Fig. S7G-I).

## Discussion

### EVs as messengers or as cellular disposal mechanisms

We have conducted a thorough interrogation of the long RNA content of endothelial cells and their released EVs in basal conditions, during activation with VEGF, and during exposure to tumor cells. We found that EVs contain long RNA transcripts, at least some of which are full-length mRNA and lncRNA, at levels that largely reflect cellular abundance. However, we also observed that the EV transcriptome is not a mere reflection of the cellular transcriptome. Indeed, many transcripts are specifically enriched or depleted in EVs relative to cells. The EV-enriched mRNA and lncRNA transcripts are on average shorter and more exon-dense than their cell-retained counterparts. They also tend to be more GC-rich and are enriched for motifs that can be bound by certain RBPs that may be involved in RNA transcript sorting. The most unexpected finding of this study is that, when cells are subjected to a stimulus, intracellular changes in mRNA and lncRNA abundance are anticorrelated with EV packaging efficiencies. Genes that are upregulated in cells in response to extracellular stimuli tend to be less packaged into EVs, while those that are downregulated in cells tend to be more exported into EVs. This raises the enticing possibility that in concert with transcriptional and post-transcriptional regulation, packaging into EVs can contribute to the control of intracellular RNA levels.

In recent years, research in the field of extracellular vesicles has focused heavily on the role of EVs as intercellular messengers, carrying functional RNA and protein cargos to distant cells and tissues. While many groups have established that exogenous EVs can impact recipient cells, less attention has been paid to the impact of EV production on parent cells. Interestingly, early studies cast EVs as a disposal mechanism, in particular removing proteins whose functions are no longer required as cells proceed through developmental stages [51, 52]. More recently, Takahashi et al. have demonstrated a possible role for EVs in discarding harmful cytoplasmic DNA from cells [53]. Likewise, Teng et al. showed that colon cancer cells use EVs to expel the tumor-suppressor miR-193a [54]. Supporting this notion, artificially decreasing cellular miRNA levels with an inducible Dicer knockout reduced miRNA levels in EVs even more strongly than in cells, possibly reflecting decreased miRNA packaging into EVs as the cells struggled to regain homeostasis. Likewise, overexpressing a miR led to more exaggerated overexpression in EVs than in cells [55]. Interestingly, this phenomenon may extend to exogenous materials present in cells as well, as illustrated for prostate cancer cells that can secrete the anti-cancer drugs docetaxel and methotrexate in large EVs. When EV release is inhibited in these cells, the drugs accumulate intracellularly and drug-induced apoptosis increases [56]. Our evidence extends this role for EVs as a disposal mechanism for un-needed or hazardous molecules to include long RNA. We observed an inverse correlation between EV packaging and cellular transcript abundance for both mRNA and lncRNA; it is possible that this mechanism extends to other RNA species as well. While our RNA-Seq library and LoTEVA pipeline are designed to assess long RNAs, smaller species including miRNAs, piRNAs, vault RNAs, and others are present in EVs [1], and future studies aimed at addressing this issue can test conditions that lead to altered cellular abundance of these transcripts and investigate their changing levels in both cells and EVs.

### Export in EVs adds to the cellular arsenal controlling RNA intracellular homeostasis

The regulation of RNA transcript abundance in the cell is generally thought of as a balance between transcription and decay. Our evidence indicates that export of long coding and noncoding RNA transcripts in EVs is a potential third mechanism to control intracellular abundance. Notably, RNA-Seq coverage indicated that long RNA transcripts in EVs tend to be spliced (Fig. 3G, H, Additional file 4: Fig. S3C-G). This suggests that mature, translation-competent mRNA transcripts are packaged from the cytoplasm into EVs, implicating EV packaging as a level of control that could supplement the effects of transcriptional downregulation by acting on transcripts that have already been exported from the nucleus. Interestingly, we observe that EVs are enriched for shorter mRNA and lncRNA transcripts, transcripts that are more highly spliced, and mRNA transcripts coding for ribosomal proteins; all characteristics that are associated with more stable RNA [32, 33, 57]. Indeed, using RNA half-life information estimated from 4sU-labeling and RNA-Seq of lymphoblastoid cell lines [34, 35], from both 5′-bromo-uridine immunoprecipitation chase–deep sequencing (BRIC-seq) [36, 37] and RNA-Seq following actinomycin-D treatment of HeLa cells (Fig. 3G) and from RNA-Seq following actinomycin-D treatment of A673 cells (Additional file 4: Fig. S3C), we observed that long RNA transcripts enriched in EVs tend to have longer half-lives than those transcripts preferentially retained in cells (Fig. 3G–I). While the relative contribution of different RNA disposal mechanisms remains to be elucidated, packaging into EVs may represent an effective removal strategy for the shorter, more stable transcripts that present greater resistance to canonical RNA decay pathways. The interplay of transcription, decay, and secretion is likely very complex and further investigation will be necessary to decipher control mechanisms.

### Mechanisms of EV sorting

In addition to stability-related sequence features, we identified enrichment in binding motifs for the RBP HNRNPA2B1 in EV-enriched long RNA transcripts. This protein has previously been shown to control the sorting of miRNA transcripts into EVs in a variety of cell types. Data from our siRNA knockdown experiments suggest that HNRNPA2B1 might also participate in sorting mRNA and lncRNA into EVs. The most straightforward model involves the protein and its bound RNA transcripts being incorporated into EVs in response to some signal and indeed, the HNRNPA2B1 protein has been detected previously in EVs [39]. Others, however, have reported that the HNRNPA2B1 protein is not present in EVs [58], and HNRNPA2B1-dependent mechanisms that do not involve its presence in EV are certainly possible [58]. For example, given the high density of splice junctions in EV-enriched transcripts, one could speculate that HNRNPA2B1, a reader of RNA methylation and regulator of splicing, could “mark” transcripts for EV packaging via some modification of the exon-junction complexes that are deposited on spliced RNA. Such a system could target transcripts to EV packaging even after HNRNPA2B1 is no longer bound to the transcript. This RBP-based sorting mechanism could ensure high specificity, as illustrated by the distinct effects observed by knocking down HNRNPA2B1, but not its close relative HNRNPA1 (Fig. 7C). Notably however, many EV-enriched transcripts do not appear to be bound by HNRNPA2B1, and other RBPs are likely involved.

#### mRNA and lncRNA sorting mechanisms

We see strikingly similar behavior between mRNA and lncRNA transcripts in our study. Examined separately and together, mRNA and lncRNA show similar levels of anticorrelation between EV packaging rates and cellular abundance changes in changing cellular conditions (Figs. 5 and 6C, D). Further, both mRNA and lncRNA show a strong correlation between cellular and EV abundance (Fig. 2B, C) and appear to be present as full-length transcripts in many cases (Fig. 2E, F). The numbers of lncRNAs expressed in the cell, and preferentially exported to EVs, are smaller than the corresponding numbers of mRNAs, which limits statistical power in assessing different characteristics. Still, we were able to observe that EV-enriched transcripts of both mRNA and lncRNA tend to be shorter and more exon-dense than mRNA and lncRNA transcripts that are preferentially retained in cells (Fig. 3A, Additional file 4: Fig. S3C-I), and also have a higher G/C content (Additional file 5: Fig. S4) and are more likely to contain binding sites associated with HNRNPA2B1 (Fig. 4). In terms of EV packaging, antisense lncRNAs appear to be regulated independently of their opposing protein-coding genes (Additional file 4: Fig. S3A), and intergenic lncRNAs appear to be regulated independently of their nearest-neighbor protein-coding genes (Additional file 4: Fig. S3B). There is yet not a complete consensus in the literature as to whether the transcription and decay of lncRNA genes as a class are regulated by the same mechanisms as protein-coding genes [59, 60]. The evidence we present here of shared structural and sequence characteristics of EV-enriched mRNAs and lncRNAs suggests that mRNA and lncRNA genes are similarly regulated in terms of packaging into EVs.

### Subsets of EVs

There is growing evidence that many different subsets of EVs are produced, even by the same cell or tissue type. Classes of EVs of different sizes and with different protein markers can be identified within single samples (e.g., see ref. [3]), and different biogenesis pathways have been identified even for EVs with comparable physical characteristics. For example, both a syntenin (SDCBP)/syndecan-associated, ESCRT-dependent pathway [50] and a ceramide-dependent, ESCRT-independent pathway [48] have been identified as contributing to the production of small, ALIX-enriched, endosomal-derived EVs. We found that separately inhibiting these distinct pathways led to different impacts on cellular levels of transcripts typically highly exported to EVs. For example, when we treated cells with GW4869 to inhibit ceramide-dependent EV biogenesis, we saw increases in the cellular level of HDAC5 transcripts that we did not observe when we treated cells with Src Inhibitor 1 to inhibit syntenin (SDCBP)/syndecan-associated EV biogenesis. Further, when we treated cells with ketoconazole, an EV biogenesis inhibitor of unknown mechanism, we obtained still different results (Fig. 4C). Electron microscopy showed that the EV samples we prepared to identify EV-enriched and depleted transcripts appeared, as expected, to be a mixed populations of multiple different EV subsets (Additional file 1: Fig. S1C). The different responses to EV-inhibitor compounds strongly imply that EV subsets contain distinct RNA profiles. This raises exciting possibilities for new lines of investigation into the selection and packaging of specific transcripts into distinct EV subsets. The roles of transcript sequence and structure, RBPs and EV biogenesis pathways, and their interrelationships remain to be assessed in the context of heterogeneous mixtures of EVs.

### Implications for EV research

While transcript abundance in EVs is strongly correlated with transcript abundance in cells, our results indicate that EV contents should not necessarily be regarded as perfect “snapshots” of cellular contents, especially in dynamic contexts in which cells are reacting to changing environmental stimuli. Cells responding to altered environments might package surprisingly high or low amounts of specific transcripts, depending on intracellular needs. Our findings thus have implications for EV study design and in particular for the interpretation of EVs as biomarkers.

## Conclusions

While recent EV research has emphasized the role of EVs as messengers that affect the behavior of distant recipient cells, our study suggests a role of EVs in regulating the transcriptome of the parent cell. This is consistent with the hypotheses of early EV research with regard to protein EV cargos, as well as more recent evidence for DNA and miRNAs. Importantly, this model does not exclude a messenger function for EVs: indeed, we have also observed functional impacts on recipient cells treated with endothelial cell EVs (manuscript in preparation). However, our evidence indicates that the “messages” packaged into EVs should also be considered in the context of their contribution to the parent cell, in particular as part of the complicated machinery regulating cellular homeostasis and response to environmental change.

## Methods

### Cell culture

Human Umbilical Vein Endothelial Cells (Lonza) were amplified in a 37°C incubator with 5% CO2 in endothelial growth media-2 (EGM-2, Lonza) with 5% FBS and 5 ng/ml rhFGF (Promega) and without heparin. Cells between passages 2 and 8 were used for all experiments. When indicated, cells were treated with 5 μM GW4869 (Sigma-Aldrich D1692), 5 μM Src Inhibitor 1 (Sigma-Aldrich S2075), 10 μM ketoconazole (Sanbio 15212-100), or an equivalent volume of DMSO for 48 h, with fresh drug or DMSO added after 24 h. When indicated, cells were treated with 100 ng/ml recombinant human VEGF165 (Peprotech 100-20) or an equal volume of water for 48 h. MDA-MB-231 cells were maintained in a 37°C incubator with 5% CO2 in DMEM with 5% FBS.

### EV isolation

Exosome-depleted FBS was prepared by centrifuging FBS, diluted 50% with EGM-2, in an SW 32 Ti swinging-bucket rotor in an ultracentrifuge at 110,000×*g* for at least 16 h. Twenty-four hours before exosome collection, cells were rinsed with DPBS and placed in EGM-2 media with 1% exosome-depleted FBS and 5 ng/ml rhFGF and without heparin. Cells were then rinsed with DPBS and the media replaced with fresh EGM-2 media with 1% exosome-depleted FBS, 5 ng/ml rhFGF, and without heparin for EV collection. After 48 h, the conditioned media was harvested and EVs enriched via an ultracentrifugation protocol (slightly modified from Théry et al. 2006 [61]): conditioned media were spun in a centrifuge at 400×*g* for 5 min to pellet any cells and cell debris. Supernatant was removed and spun in a centrifuge for 2000×*g* for 20 min at 4°C, then supernatant was again removed and spun in a centrifuge at 12,000×*g* for 45 min at 4°C. Supernatant was removed and filtered through a 0.22-μm filter (Millipore SCGP00525), then spun in an SW 32 Ti swinging-bucket rotor in an ultracentrifuge at 110,000×*g* for 120 min. The supernatant was removed and the EV pellet resuspended in PBS. EV size distributions were obtained using dynamic light scattering (DLS) on a Zetasizer Nano ZS instrument. To obtain protein concentrations EVs were lysed with lysis buffer (10% Triton, 1% SDS in PBS) and protein concentrations determined using a Piece BCA Protein Assay kit (ThermoFisher #23225) with a 60-min incubation at 60°C.

### Cell coculture

Twenty-four hours before initiating coculture, HUVEC cells were rinsed with DPBS and placed in EGM-2 media with 1% exosome-depleted FBS and 5 ng/ml rhFGF and without heparin, and MDA-MB-231 cells were rinsed with DPBS and placed in DMEM with 5% exosome-depleted FBS. To initiate coculture, MDA-MB-231 cells were treated with trypsin, rinsed twice in DPBS and resuspended in EGM-2 media with 1% exosome-depleted FBS and 5 ng/ml rhFGF and without heparin. HUVEC cells were rinsed twice in DPBS and placed in EGM-2 media with 1% exosome-depleted FBS and 5 ng/ml rhFGF and without heparin. MDA-MB-231 cells were added to plates of adherent HUVEC cells in a 1:1 ratio of near-confluent plates. EVs were purified from the conditioned media after 48 h.

To purify HUVEC cells and their EVs from mixed populations, Dynabeads CD31 Endothelial Cell magnetic beads (Invitrogen 11155D) were used. Cells were purified according to the manufacturer’s instructions. CD31+ EVs were purified by resuspending the ultracentrifugation EV pellet in Dynabeads Isolation buffer, adding to 50 μl of washed beads per T-175 of culture, incubating overnight at 4°C with rotation, washing twice with isolation buffer, then resuspending beads with attached EVs in lysis buffer or PBS as required for downstream applications.

### RNA isolation

RNA was isolated from cells and EVs using the Nucleospin RNA kit (Macherey Nagel) according to the manufacturer’s protocol and quantified with a Nanodrop instrument (Thermo Scientific). For monoculture and coculture experiments, RNA was isolated from cells and EVs using the miRNeasy kit (QIAGEN).

### Western blot

Cells were lysed in RIPA buffer (50 mM Tris pH 7.5, 150 mM NaCl, 10 mM CaCl2, 0.5% NP40, 0.25% sodium deoxycholate and 0.1% SDS) and EVs were lysed in lysis buffer (10% Triton, 1% SDS in PBS). Samples in loading buffer (40% Glycerol, 240 mM Tris/HCl pH 6.8, 8% SDS, 0.025% Bromophenol Blue) were heated to 100°C for 10 min. Ten micrograms of protein per sample was loaded and electrophoresed on an SDS-acrylamide gel, then transferred to a nitrocellulose membrane. Membranes were blocked with milk and stained with primary antibodies: PDCD6IP (ALIX; Abcam 186429), SDCBP (syntenin; Abcam 133267), CD9 (Santa Cruz SC20048), HSP70 (Santa Cruz SC33575 [H-300]), tubulin (Abcam 6046), HNRNPA1 (Sigma R4528), and hnNRPA2/B1 (Abcam 6102 [DP383]), then incubated with appropriate secondary antibodies coupled to horseradish peroxidase (Santa Cruz). Blots were developed using chemiluminescence.

### Cryo-transmission electron microscopy (Cryo-TEM)

EVs were visualized by the Cryo-TEM method. A 3-μl droplet of each EV suspension was applied to a glow-discharged holey carbon grid (Lacey Carbon Grids). After the application of the suspension, the grid was blotted against filter paper, leaving a thin sample film spanning the grid holes. These films were vitrified by plunging the grid into ethane, which was kept at its melting point by liquid nitrogen, using a Vitrobot (Thermo Fisher) to keep the sample at 95% humidity before blotting and freezing. The vitreous sample films were transferred to a Tecnai Arctica microscope (Thermo Fisher). Images were taken at 200 Kv with a field emission gun using a Falcon III (Thermo Fisher) direct electron detector.

### RNA-Seq

RNA size profiles and were determined with a Bioanalyzer instrument. For experiments comparing EVs to cells, ribodepleted RNA-Seq libraries were prepared with the Ovation SoLo Human RNA-Seq system (NuGen) using 10 ng RNA as input. For EV inhibitor experiments, RNA-Seq libraries were prepared with the TruSeq Stranded mRNA kit (Illumina) using 1 μg RNA as input. Libraries were sequenced as 75-nt single end reads on an Illumina NextSeq500 instrument. *N* = 3 for all RNA-Seq experiments except monoculture and coculture cells, for which *N* = 4. Read depth is indicated in Table 1.

**Table 1:** Read depth

Cell treatment	Sample	Read depth
None	Cells	1.2–1.3 × 10^7^
	EVs	3.5–3.8 × 10^7^
VEGF	Cells	1.4–1.5 × 10^7^
	EVs	3.5–4.1 × 10^7^
Monoculture	Cells	2.0–2.2 × 10^7^
	EVs	2.5–2.7 × 10^7^
Coculture	Cells	2.1–2.3 × 10^7^
	EVs	2.5–3.1 × 10^7^
None	Cells	1.9–2.0 × 10^7^
GW4869	Cells	1.9–2.0 × 10^7^

### RNA-Seq analysis

Ovation SoLo RNA-Seq reads were trimmed of their first 5 nucleotides as per vendor protocol, and low complexity reads were removed using Prinseq. For genome-wide visualization of RNA-Seq coverage, reads were aligned and mapped to version GRCh38 of the human genome with STAR aligner V. 2.5.2b and visualized with J-Circos. For quantification, reads were first mapped to the set of NCBI RefSeq rRNA sequences using STAR aligner V. 2.5.2b. Unmapped reads were then mapped to the GRCh38.90 human transcriptome from Ensembl (cDNA + noncoding RNA) using Salmon v.0.8.2 [62]. Reads that were likely PCR duplicates were removed using an in-house Python script that identified reads with matching Ovation SoLo barcodes that mapped within 38 bp of each other. Finally, remaining reads were once again mapped to the GRCh38.90 human transcriptome using Salmon v.0.8.2. Genes with an abundance level of at least 1 transcript per million (TPM) averaged across the replicates were considered to be reliably detected. To investigate differential gene expression, mRNA and lncRNA read counts were summed to the gene level using tximport and compared using DESeq2 [63]. Reads mapping to genes with the Ensembl gene biotypes *protein_coding* or *translated_processed_pseudogene* were considered to arise from mRNA. Reads mapping to genes with following Ensembl gene biotypes were considered to arise from lncRNA: *lincRNA*, *antisense_RNA*, *bidirectional_promoter_lncRNA*, *macro_lncRNA,non_coding*, *processed_transcript*, *sense_intronic*, *sense_overlapping*, *3prime_overlapping_ncRNA*, *polymorphic_pseudogene*, *processed_pseudogene*, *pseudogene*, *transcribed_processed_pseudogene*, *transcribed_unitary_pseudogene*, *transcribed_unprocessed_pseudogene*, *unitary_pseudogene*, and *unprocessed_pseudogene.* Change in EV-packaging upon treatment with VEGF or tumor exposure was calculated as:

$$\mathrm{log}2\ \left(\mathrm{EV}-\mathrm{cell}\ \mathrm{fold}\ \mathrm{difference}\ \mathrm{in}\ \mathrm{experimental}\ \mathrm{condition}\right)-\mathrm{log}2\ \left(\ \mathrm{EV}-\mathrm{cell}\ \mathrm{fold}\ \mathrm{difference}\ \mathrm{in}\ \mathrm{basal}\ \mathrm{condition}\right)$$

Functional enrichment analysis was performed using GSEA MSigDB [64] hallmark gene sets (pre-ranked analysis of protein-coding genes by fold change, using classic enrichment statistic). Lists of differentially expressed genes (padj < 0.1, TPM >= 1) were also analyzed with DAVID [65, 66]. Comparisons of feature lengths, exon density, percent GC, and ARE motif abundance, as well as motif enrichment analysis, were performed using the primary transcript (determined by APPRIS [67], or the longest transcript) of each gene. Motif enrichment analysis was performed with AME [38] against the Ray [68] RBP motif dataset using Fisher’s exact test.

RNA-Seq read coverage of transcripts was determined by first selecting a primary transcript for each gene using the APPRIS annotation or, for genes without APPRIS annotation, the longest annotated transcript. For mRNA, for each primary transcript, the most 5′-mapped and most 3′ mapped reads within the coding sequence were located, and the distance between these transcripts was divided by the total coding sequence length. For lncRNA, for each primary transcript, the most 5′-mapped and most 3′ mapped reads within the transcript were located, and the distance between these transcripts was divided by the total transcript length. Because read depth was higher for cell samples than for EV samples, reads were randomly selected from cell samples prior to mapping so that the same number of reads were mapped for cells and for EVs. For each transcript, coverage was averaged across the 3 replicates and plotted.

TruSeq Stranded mRNA reads were processed with Prinseq to remove low-complexity reads and mapped to the set of NCBI RefSeq rRNA sequences using STAR aligner V. 2.5.2b. Reads that did not map to rRNA were mapped to the GRCh38.90 human transcriptome from Ensembl (cDNA + noncoding RNA) using Salmon v.0.8.2. To investigate differential gene expression, mRNA and lncRNA read counts were summed to the gene level using tximport and compared using DESeq2.

In-house Python scripts and R scripts using packages such as BiomaRt, dplyr, pheatmap, and ggplot2 were used for analysis and visualization of data.

### PCR

For qRT-PCR, RNA was reverse-transcribed using the RevertAid H Minus First Strand cDNA Synthesis Kit (Thermo Fisher), then amplified and quantified using FastStart SYBR Green Master mix (Roche) on a LightCycler 480 instrument (Roche). Fold differences were calculated using the Δ ΔCt method. To compare abundance in cells and EVs, Ct differences were normalized to TMSB4X, a gene not significantly different between cells and EVs (log2(Fold Difference) = 0.34, padj = 0.52, Additional files 3, 7, 10, 11). For EV inhibitor experiments, Ct differences were normalized to the average of TCIRG1 and HOOK2, two genes that are highly cell-enriched and nearly undetectable in EVs, and that are unchanged in RNA-Seq data upon treatment with GW4869 (TCIRG1 log2(Fold Change) = −0.19, padj = 0.46, HOOK2 log2(Fold Change) = 0.03, padj = 0.98, Additional files 3, 7, 8, 10, 11, 12, 16). For si*HNRNPA2B1* and si*HNRNPA1* experiments, Ct differences were normalized to GPR107, which does not have recognizable HNRNPA2B1 or HNRNPA1 binding motifs, was not identified as an HNRNPA2B1 binding target by HITS-CLIP [43, 44], is highly cell-enriched, and is nearly undetectable in EVs. Primers used are shown in Table S1.

For whole-transcript PCR, RNA was reverse-transcribed using the RevertAid H Minus First Strand cDNA Synthesis Kit (Thermo Fisher). Dilutions (10 to 100 fold depending on the transcripts) of the cDNA reactions were amplified using iTaq (Bio-Rad) according the manufacturer’s instructions using 36–40 cycles (30 s at 95°C, 30 s at 55–60°C and 1min/kb at 72°C with a final step of 5 min at 72°C). PCR products were electrophoresed on an acrylamide gel with ethidium bromide. Primers used are shown in Table S1.

### siRNA

For siRNA transfection, 350,000 HUVEC were seeded per well of a 6-well plate 24 h prior to transfection. GeneTrans II transfection reagent (MoBiTech) was used to transfect 100 pmol of siRNA (50 nM final) according to vendor protocol, with a PBS wash and media change after 4 hours to prevent toxicity. Cells were harvested 48 hours after transfection. The sequences of siRNA used were si*HNRNPA2B1* #1 GGUGGCUUAAGCUUUGAAAdTdT, si*HNRNPA2B1* #2 GGAACAUCACCUUAGAGAUUACUdTdT [40], and si*HNRNPA1* CAGCUGAGGAAGCUCUUCAdTdT [69] (all from Eurogentec). Control siRNA was purchased from Eurogentec (Control siRNA duplex negative control - 5 nmol; SR-CL000-005).

### Identification of HNRNPA2B1 binding targets

The sequences of the primary transcripts (determined by APPRIS [67]) of EV-enriched and EV-depleted genes were analyzed for the presence of UAG-containing motifs (RNCMPT00024, RNCMPT00022, RNCMPT00023, RNCMPT00041, RNCMPT00049) and GGAG-containing motifs (RNCMPT00036, RNCMPT00162, RNCMPT00154) using FIMO [70]. Also, EV-enriched and EV-depleted genes were examined for the presence of HNRPA2B1 binding peaks as determined in a previous HITS-CLIP analysis [43, 44].

## Supplementary Information

**Additional file 1: Figure S1.** Validation of extracellular vesicle isolation. (A) Representative western blot of cell and EV lysates using indicated antibodies. (B) DLS assessment of particle sizes in representative EV-enriched sample (C) TEM images showing vesicles in representative EV-enriched sample. Scale bar = 100 nm (D) Bioanalyzer traces of total RNA extracted from representative EV-enriched sample (top) and RNA-Seq library made from EV RNA, with fragments >200 nt amplified (bottom).

**Additional file 2: Figure S2.** (A) Plot of RNA-Seq reads from 3 EV and 3 cell samples that map to more than one genomic location. Reads mapping to rRNA are excluded. Individual values can be found in Additional file 17. (B) Hierarchical clustering of long RNA transcripts by abundance determined by RNA-Seq in 3 EV and 3 cell samples. Data are read counts transformed using the Variance Stabilizing Transformation, top 200 transcripts with the highest variance across samples are displayed. (C) RNA-Seq read coverage (top) and RT-PCR amplicons (bottom) of HNRNPA1 (D), ANP32B (E), RPL14 (F) and RPL41 (F) mRNA. (G) RNA-Seq read coverage (top) and RT-PCR amplicons (bottom) of lncRNA GAS5. NT = no template, no-RT = RNA without reverse transcriptase. Uncropped gel images can be found in Additional file 18.

**Additional file 3. **Fold changes, adjusted *p*-values and TPMs for genes in cells and EVs.

**Additional file 4: Figure S3.** (A) EV enrichment/depletion of expressed antisense (AS) genes and their protein-coding (PC) complements. Red = significantly enriched or depleted antisense gene. Blue = significantly enriched or depleted protein-coding gene. Purple = both antisense gene and protein-coding gene are significantly enriched/depleted. Padj < 0.1 for significance. *N* = 5526 expressed protein-coding/antisense gene pairs. (B) EV enrichment/depletion of expressed long intergenic noncoding RNA (lincRNA) genes and the nearest protein-coding genes. Red = significantly enriched or depleted lincRNA. Blue = significantly enriched or depleted protein-coding gene. Purple = both lincRNA and neighboring protein-coding gene are significantly enriched/depleted. Padj < 0.1 for significance. *N* = 7592 expressed lincRNA/neighboring protein-coding gene pairs. (C—F) Violin plots of mRNA transcript length (C), 5’UTR length (D), CDS length (E), 3’UTR length (F) for EV-enriched (*n* = 609) and EV-depleted (*n* = 680) protein-coding genes. (G) Violin plot of lncRNA transcript length for EV-enriched (*n* = 72) and EV-depleted (*n* = 123) lncRNA genes. (H-I) Violin plots of number of exons per kilobase of transcript length for EV-enriched and EV-depleted protein-coding (H) or lncRNA genes (I). (J) Violin plot of transcript half-life in 4SU-labeled LCLs for EV-enriched and EV-depleted transcripts (K) Violin plot of transcript half-life in HeLa cell BRIC-Seq for EV-enriched and EV-depleted transcripts (L) Violin plot of transcript half-life in Actinomycin D-treated A673 cell RNA-Seq for EV-enriched and EV-depleted transcripts. For all violin plots, medians are indicated above each violin and grey dotted lines indicate median of all expressed genes. *P*-values calculated by Welch two-sample t-test are indicated. (M-N) Plots of occurrences of ARE elements per kilobase of transcript in 3’ UTRs (M) and 5’ UTRs (N) of EV-enriched and EV-depleted transcripts. Individual values can be found in Additional file 17. All analyses were performed using 3 EV and 3 cell samples. All *p*-values for differences are calculated by Welch two-sample t-test.

**Additional file 5: Figure S4.** (A) Violin plots of percent GC in EV-enriched (*n* = 681) and EV-depleted (*n* = 803) mRNA and lncRNA transcripts combined (A), mRNA transcripts alone (B; *n* = 609 EV-enriched, *n* = 680 EV-depleted) and lncRNA transcripts alone (C; *n* = 72 EV-enriched, *n* = 123 EV-depleted). Median is indicated above each violin. *P*-value calculated by Welch two-sample t-test is indicated. Grey dotted line indicates median of all expressed genes. All analyses were performed using 3 EV and 3 cell samples.

**Additional file 6: Figure S5.** (A) mRNA and (B) lncRNA abundance from RNA-Seq in VEGF-treated cells and their EVs. TPM = transcripts per million. TPM values are averaged across 3 replicates. (C) Gene Ontology analysis of genes altered in cells by VEGF treatment. For each GO category the ten significant (FDR < 0.05) terms with the lowest *p*-values are displayed. BP = Biological Process, CC = Cellular Component, MF = Molecular Function. Individual values can be found in Additional file 17. (D) Volcano plot of log2 fold changes by RNA-Seq of mRNA and lncRNA genes (combined) in EVs derived from VEGF-treated cells vs. EVs derived from untreated cells. (E) Volcano plot of log2 fold changes by RNA-Seq of mRNA and lncRNA genes in VEGF-treated cells vs. untreated cells. All analyses were performed using 3 EV and 3 cell samples.

**Additional file 7. **Fold changes, adjusted *p*-values and TPMs for genes in VEGF-treated cells and their EVs.

**Additional file 8. **Fold changes, adjusted *p*-values and TPMs for genes in cells treated with VEGF or not.

**Additional file 9. **Fold changes, adjusted *p*-values and TPMs for genes in EVs obtained from cells treated with VEGF or not.

**Additional file 10. **Fold changes, adjusted *p*-values and TPMs for genes in CD31-selected cells and EVs.

**Additional file 11. **Fold changes, adjusted *p*-values and TPMs for genes in tumor-exposed, CD31-selected cells and their EVs.

**Additional file 12. **Fold changes, adjusted *p*-values and TPMs for genes in CD31-selected cells exposed to tumor cells or not.

**Additional file 13. **Fold changes, adjusted *p*-values and TPMs for genes in CD31-selected EVs obtained from cells exposed to tumor cells or not.

**Additional file 14: Figure S6.** (A) Gene Ontology analysis of protein-coding genes altered in cells by coculture. For each category the top 10 most significantly enriched GO terms (FDR < 0.05) are displayed. BP = Biological Process, CC = Cellular Compartment, MF = Molecular Function. Individual values can be found in Additional file 17. (B) Volcano plot of log2 fold changes by RNA-Seq of mRNA and lncRNA genes (combined) in EVs derived from tumor-exposed cells vs. EVs derived from unexposed cells. (C) Log2 fold change in tumor-exposed cells vs. unexposed cells and in EVs derived from tumor-exposed cells vs. EVs derived from unexposed cells for the most increased (left) and decreased (right) mRNA and lncRNA genes significantly changed in cells. Error bars represent standard error of log2 fold change. Individual values can be found in Additional file 17. (D-E) Depletion of E2F_TARGETS_V1 gene set (D) and G2M_TARGETS gene set (E) in tumor-exposed vs. unexposed cells and enrichment of the same gene set in EVs derived from tumor-exposed vs. unexposed cells. All analyses were performed using 3 EV and 3 cell samples.

**Additional file 15: Figure S7.** (A) Western blot of lysate from cells treated with GW4869 or DMSO and from EVs derived from cells treated with GW4869 or DMSO, using indicated antibodies (left). (B-C) Distributions of RNA-Seq log2 fold changes of (B) protein-coding genes and (C) lncRNA enriched in EVs (green) and not enriched in EVs (purple) in cells treated with GW4869. *N* = 3. (D) Western blot of lysates from cells treated with Src Inhibitor 1 or DMSO and from EVs derived from cells treated with Src inhibitor 1 or DMSO, using antibodies to SDCBP (exosome marker) or tubulin (control). (E) Western blot of lysates from cells treated with ketoconazole or DMSO and from EVs derived from from cells treated with keotconazole or DMSO, using antibodies to SDCBP (exosome marker) or tubulin (control). (F) Enrichment in EVs vs. cells by RNA-Seq (green scale; *n* = 3) and indication of GGAG motif, UAG motif or HNRNPA2B1 CLIP peak presence in indicated transcripts. (G-I) Western blots of lysates from cells treated with control siRNA (siCTRL) in comparison with cells treated with (G) si*HNRNPA2B1* #1, (H) si*HNRNPA2B1* #2 or (I) si*HNRNPA1* using indicated antibodies. Uncropped images of blots can be found in Additional file 18.

**Additional file 16. **Fold changes, adjusted *p*-values and TPMs for genes in cells treated with GW4869 or not.

**Additional file 17.** Raw data for graphs with N < 6.

**Additional file 18.** Uncropped gels and blots.

Additional file 19: **Table S1.** PCR primers

## Data Availability

All data generated or analyzed during this study are included in this published article, its supplementary information files, and publicly available repositories. Individual values for figures with *n* < 6 can be found in Additional file 17. All uncropped gels and blots can be found in Additional file 18. The RNA-Seq datasets generated in this study are available in the GEO repository under the accession numbers GSE164868 (endothelial cells and EVs treated with VEGF or H_2_O), GSE164869 (endothelial cells and EVs cultured alone or with tumor cells), GSE164863 (endothelial cells treated with GW4869), GSE198177 (half-life determination in A673 cells), and GSE198178 (half-life determination in HeLa cells). Previously published RNA half-life data and HITS-CLIP data are available from the papers [34, 36, 43] as well as public repositories (GEO GSE34204, SRA DRR000881 and GEO GSE35799).

## Abbreviations

AME: Analysis of Motif Enrichment
ARE: AU-rich element
AS: Antisense
BRIC-Seq: 5′-Bromo-uridine immunoprecipitation chase–deep sequencing
CDS: Coding sequence
DLS: Dynamic light scattering
EVs: Extracellular vesicles
GO: Gene Ontology
HUVEC: Human umbilical vein endothelial cells
lincRNA: Long intergenic noncoding RNA
lncRNA: Long noncoding RNA
LoTEVA: Long Transcript EV Abundance
PC: Protein-coding
RBP: RNA-binding protein
TPM: Transcripts per million
VEGF: Vascular endothelial growth factor
