# Supplementary material for: Sorting and packaging of RNA into extracellular vesicles shape intracellular transcript levels
Source: BMC Biol. 2022 Mar 24;20:72. doi: 10.1186/s12915-022-01277-4 (PMC8944098; doi:10.1186/s12915-022-01277-4)
Supplement: Supplementary file 1 — Additional file 1: Figure S1. Validation of extracellular vesicle isolation. (A) Representative western blot of cell and EV lysates using indicated antibodies. (B) DLS assessment of particle sizes in representative EV-enriched sample (C) TEM images showing vesicles in representative EV-enriched sample. Scale bar = 100 nm (D) Bioanalyzer traces of total RNA extracted from representative EV-enriched sample (top) and RNA-Seq library made from EV RNA, with fragments >200 nt amplified (bottom). [file 12915_2022_1277_MOESM1_ESM.pdf]

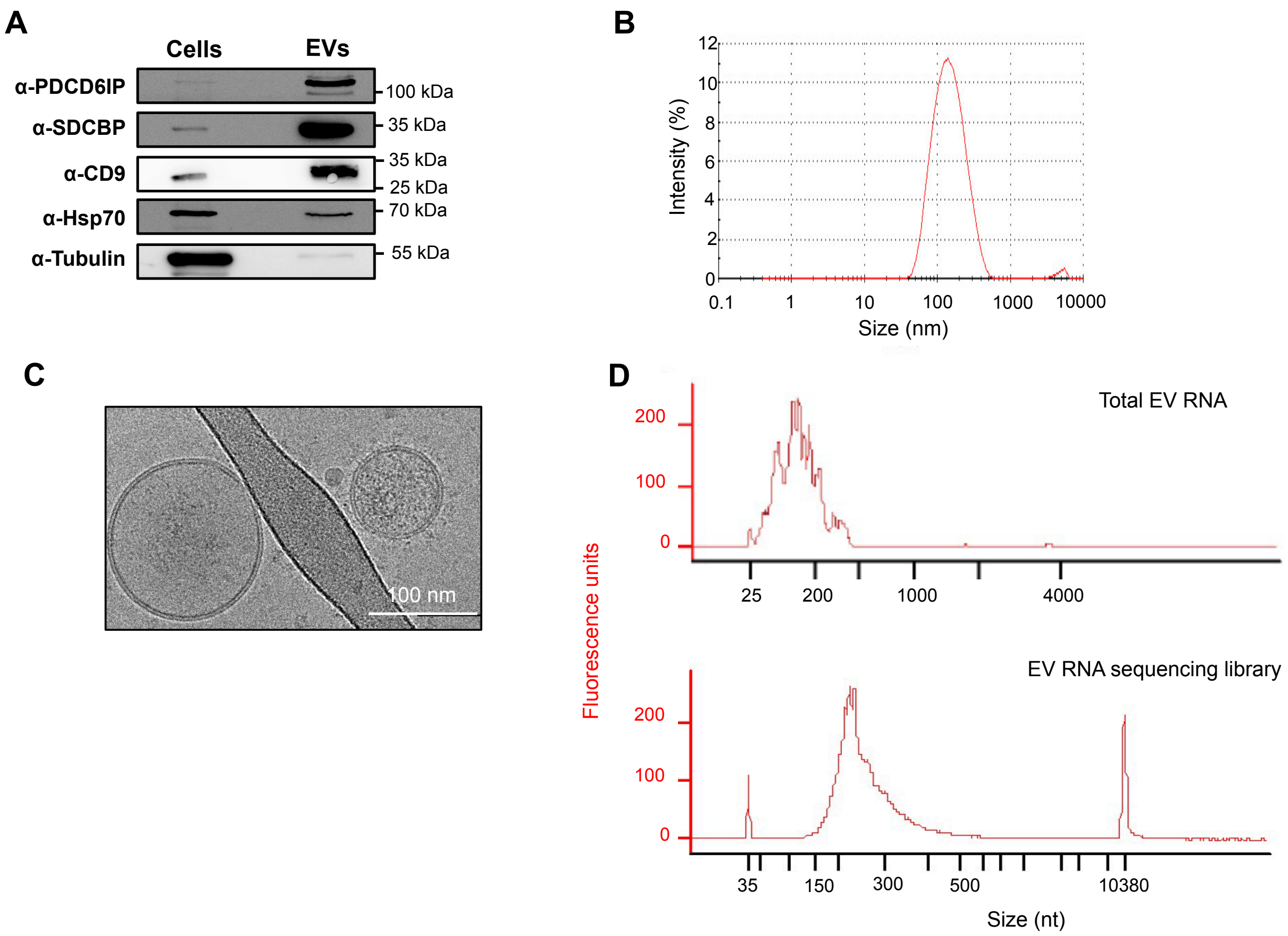

Figure S1: Validation of extracellular vesicle isolation. (A) Representative western blot of cell and EV lysates using indicated antibodies. (B) DLS assessment of particle sizes in representative EV-enriched sample (C) TEM images showing vesicles in representative EV-enriched sample. Scale bar = 100 nm (D) Bioanalyzer traces of total RNA extracted from representative EV-enriched sample (top) and RNA-Seq library made from EV RNA, with fragments >200 nt amplified (bottom)
