# Supplementary material for: Sorting and packaging of RNA into extracellular vesicles shape intracellular transcript levels
Source: BMC Biol. 2022 Mar 24;20:72. doi: 10.1186/s12915-022-01277-4 (PMC8944098; doi:10.1186/s12915-022-01277-4)
Supplement: Supplementary file 2 — Additional file 2: Figure S2. (A) Plot of RNA-Seq reads from 3 EV and 3 cell samples that map to more than one genomic location. Reads mapping to rRNA are excluded. Individual values can be found in Additional file 17. (B) Hierarchical clustering of long RNA transcripts by abundance determined by RNA-Seq in 3 EV and 3 cell samples. Data are read counts transformed using the Variance Stabilizing Transformation, top 200 transcripts with the highest variance across samples are displayed. (C) RNA-Seq read coverage (top) and RT-PCR amplicons (bottom) of HNRNPA1 (D), ANP32B (E), RPL14 (F) and RPL41 (F) mRNA. (G) RNA-Seq read coverage (top) and RT-PCR amplicons (bottom) of lncRNA GAS5. NT = no template, no-RT = RNA without reverse transcriptase. Uncropped gel images can be found in Additional file 18. [file 12915_2022_1277_MOESM2_ESM.pdf]

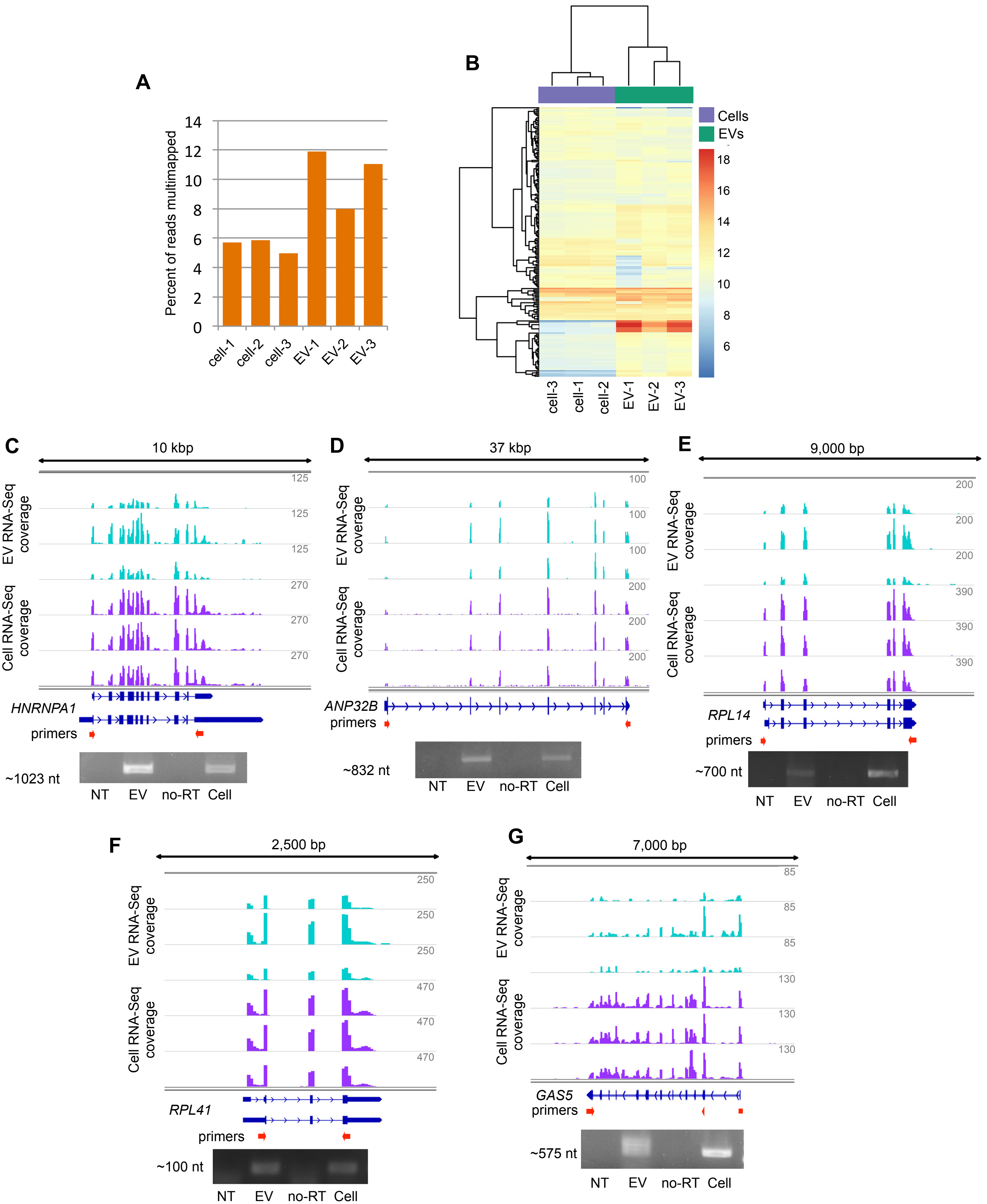

Figure S2 (legend next page)

Figure S2: (A) Plot of RNA-Seq reads from 3 EV and 3 cell samples that map to more than one genomic location. Reads mapping to rRNA are excluded. Individual values can be found in Additional file 17. (B) Hierarchical clustering of long RNA transcripts by abundance determined by RNA-Seq in 3 EV and 3 cell samples. Data are read counts transformed using the Variance Stabilizing Transformation, top 200 transcripts with the highest variance across samples are displayed. (C) RNA-Seq read coverage (top) and RT-PCR amplicons (bottom) of HNRNPA1 (D), ANP32B (E), RPL14 (F) and RPL41 (F) mRNA. (G) RNA-Seq read coverage (top) and RT-PCR amplicons (bottom) of lncRNA GAS5. NT = no template, no-RT = RNA without reverse transcriptase. Uncropped gel images can be found in Additional file 18.
