# Supplementary material for: Sorting and packaging of RNA into extracellular vesicles shape intracellular transcript levels
Source: BMC Biol. 2022 Mar 24;20:72. doi: 10.1186/s12915-022-01277-4 (PMC8944098; doi:10.1186/s12915-022-01277-4)
Supplement: Supplementary file 4 — Additional file 4: Figure S3. (A) EV enrichment/depletion of expressed antisense (AS) genes and their protein-coding (PC) complements. Red = significantly enriched or depleted antisense gene. Blue = significantly enriched or depleted protein-coding gene. Purple = both antisense gene and protein-coding gene are significantly enriched/depleted. Padj < 0.1 for significance. N = 5526 expressed protein-coding/antisense gene pairs. (B) EV enrichment/depletion of expressed long intergenic noncoding RNA (lincRNA) genes and the nearest protein-coding genes. Red = significantly enriched or depleted lincRNA. Blue = significantly enriched or depleted protein-coding gene. Purple = both lincRNA and neighboring protein-coding gene are significantly enriched/depleted. Padj < 0.1 for significance. N = 7592 expressed lincRNA/neighboring protein-coding gene pairs. (C—F) Violin plots of mRNA transcript length (C), 5’UTR length (D), CDS length (E), 3’UTR length (F) for EV-enriched (n = 609) and EV-depleted (n = 680) protein-coding genes. (G) Violin plot of lncRNA transcript length for EV-enriched (n = 72) and EV-depleted (n = 123) lncRNA genes. (H-I) Violin plots of number of exons per kilobase of transcript length for EV-enriched and EV-depleted protein-coding (H) or lncRNA genes (I). (J) Violin plot of transcript half-life in 4SU-labeled LCLs for EV-enriched and EV-depleted transcripts (K) Violin plot of transcript half-life in HeLa cell BRIC-Seq for EV-enriched and EV-depleted transcripts (L) Violin plot of transcript half-life in Actinomycin D-treated A673 cell RNA-Seq for EV-enriched and EV-depleted transcripts. For all violin plots, medians are indicated above each violin and grey dotted lines indicate median of all expressed genes. P-values calculated by Welch two-sample t-test are indicated. (M-N) Plots of occurrences of ARE elements per kilobase of transcript in 3’ UTRs (M) and 5’ UTRs (N) of EV-enriched and EV-depleted transcripts. Individual values can be found in Additional [file 12915_2022_1277_MOESM4_ESM.pdf]

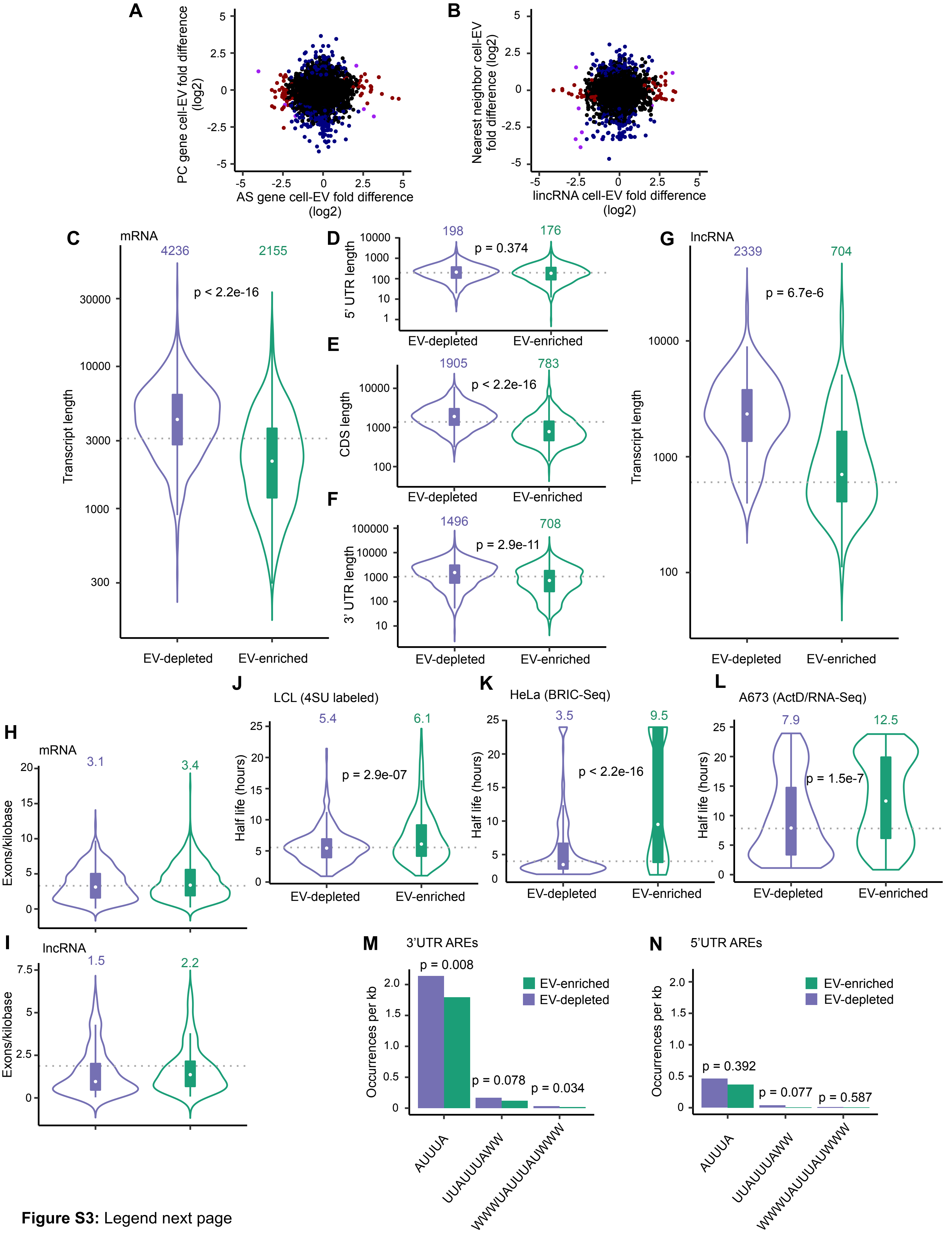

Figure S3: Legend next page

Figure S3: (A) EV enrichment/depletion of expressed antisense (AS) genes and their protein-coding (PC) complements. Red = significantly enriched or depleted antisense gene. Blue = significantly enriched or depleted protein-coding gene. Purple = both antisense gene and protein-coding gene are significantly enriched/depleted. Padj < 0.1 for significance. N = 5526 expressed protein-coding/antisense gene pairs. (B) EV enrichment/depletion of expressed long intergenic noncoding RNA (lincRNA) genes and the nearest protein-coding genes. Red = significantly enriched or depleted lincRNA. Blue = significantly enriched or depleted protein-coding gene. Purple = both lincRNA and neighboring protein-coding gene are significantly enriched/depleted. Padj < 0.1 for significance. N = 7592 expressed lincRNA/neighboring protein-coding gene pairs. (C—F) Violin plots of mRNA transcript length (C), 5'UTR length (D), CDS length (E), 3'UTR length (F) for EV-enriched (n = 609) and EV-depleted (n = 680) protein-coding genes. (G) Violin plot of lncRNA transcript length for EV-enriched (n = 72) and EV-depleted (n = 123) lncRNA genes. (H-I) Violin plots of number of exons per kilobase of transcript length for EV-enriched and EV-depleted protein-coding (H) or lncRNA genes (I). (J) Violin plot of transcript half-life in 4SU-labeled LCLs for EV-enriched and EV-depleted transcripts (K) Violin plot of transcript half-life in HeLa cell BRIC-Seq for EV-enriched and EV-depleted transcripts (L) Violin plot of transcript half-life in Actinomycin D-treated A673 cell RNA-Seq for EV-enriched and EV-depleted transcripts. For all violin plots, medians are indicated above each violin and grey dotted lines indicate median of all expressed genes. P-values calculated by Welch two-sample t-test are indicated. (M-N) Plots of occurrences of ARE elements per kilobase of transcript in 3' UTRs (M) and 5' UTRs (N) of EV-enriched and EV-depleted transcripts. Individual values can be found in Additional file 17. All analyses were performed using 3 EV and 3 cell samples. All p-values for differences are calculated by Welch two-sample t-test.
