# Supplementary material for: Sorting and packaging of RNA into extracellular vesicles shape intracellular transcript levels
Source: BMC Biol. 2022 Mar 24;20:72. doi: 10.1186/s12915-022-01277-4 (PMC8944098; doi:10.1186/s12915-022-01277-4)
Supplement: Supplementary file 5 — Additional file 5: Figure S4. (A) Violin plots of percent GC in EV-enriched (n = 681) and EV-depleted (n = 803) mRNA and lncRNA transcripts combined (A), mRNA transcripts alone (B; n = 609 EV-enriched, n = 680 EV-depleted) and lncRNA transcripts alone (C; n = 72 EV-enriched, n = 123 EV-depleted). Median is indicated above each violin. P-value calculated by Welch two-sample t-test is indicated. Grey dotted line indicates median of all expressed genes. All analyses were performed using 3 EV and 3 cell samples. [file 12915_2022_1277_MOESM5_ESM.pdf]

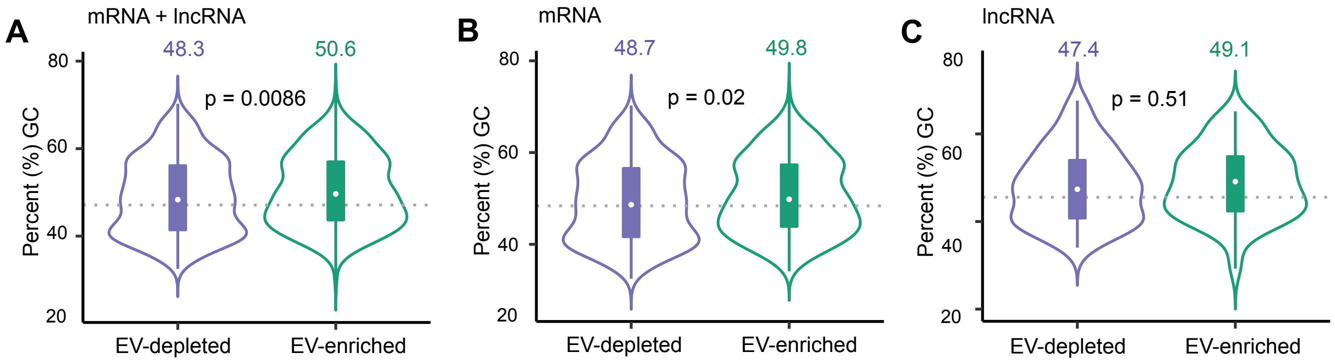

Figure S4: (A) Violin plots of percent GC in EV-enriched (n = 681) and EV-depleted (n = 803) mRNA and lncRNA transcripts combined (A), mRNA transcripts alone (B; n = 609 EV-enriched, n = 680 EV-depleted) and lncRNA transcripts alone (C; n = 72 EV-enriched, n = 123 EV-depleted). Median is indicated above each violin. P-value calculated by Welch two-sample t-test is indicated. Grey dotted line indicates median of all expressed genes. All analyses were performed using 3 EV and 3 cell samples.
