# Supplementary material for: Sorting and packaging of RNA into extracellular vesicles shape intracellular transcript levels
Source: BMC Biol. 2022 Mar 24;20:72. doi: 10.1186/s12915-022-01277-4 (PMC8944098; doi:10.1186/s12915-022-01277-4)
Supplement: Supplementary file 6 — Additional file 6: Figure S5. (A) mRNA and (B) lncRNA abundance from RNA-Seq in VEGF-treated cells and their EVs. TPM = transcripts per million. TPM values are averaged across 3 replicates. (C) Gene Ontology analysis of genes altered in cells by VEGF treatment. For each GO category the ten significant (FDR < 0.05) terms with the lowest p-values are displayed. BP = Biological Process, CC = Cellular Component, MF = Molecular Function. Individual values can be found in Additional file 17. (D) Volcano plot of log2 fold changes by RNA-Seq of mRNA and lncRNA genes (combined) in EVs derived from VEGF-treated cells vs. EVs derived from untreated cells. (E) Volcano plot of log2 fold changes by RNA-Seq of mRNA and lncRNA genes in VEGF-treated cells vs. untreated cells. All analyses were performed using 3 EV and 3 cell samples. [file 12915_2022_1277_MOESM6_ESM.pdf]

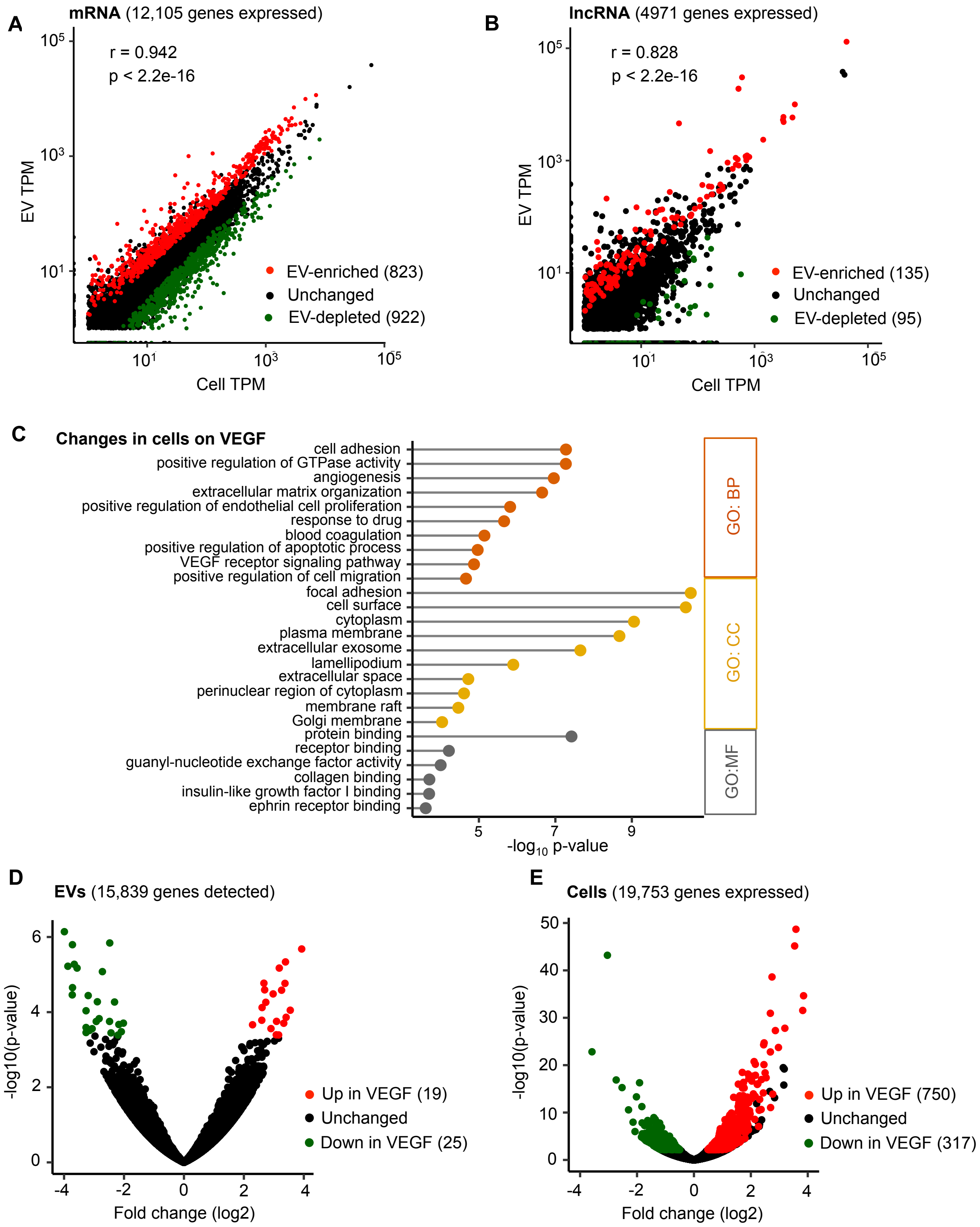

Figure S5: (A) mRNA and (B) lncRNA abundance from RNA-Seq in VEGF-treated cells and their EVs. TPM = transcripts per million. TPM values are averaged across 3 replicates. (C) Gene Ontology analysis of genes altered in cells by VEGF treatment. For each GO category the ten significant (FDR < 0.05) terms with the lowest p-values are displayed. BP = Biological Process, CC = Cellular Component, MF = Molecular Function. Individual values can be found in Additional file 17. (D) Volcano plot of log2 fold changes by RNA-Seq of mRNA and lncRNA genes (combined) in EVs derived from VEGF-treated cells vs. EVs derived from untreated cells. (E) Volcano plot of log2 fold changes by RNA-Seq of mRNA and lncRNA genes in VEGF-treated cells vs. untreated cells. All analyses were performed using 3 EV and 3 cell samples.
