# Supplementary material for: Sorting and packaging of RNA into extracellular vesicles shape intracellular transcript levels
Source: BMC Biol. 2022 Mar 24;20:72. doi: 10.1186/s12915-022-01277-4 (PMC8944098; doi:10.1186/s12915-022-01277-4)
Supplement: Supplementary file 14 — Additional file 14: Figure S6. (A) Gene Ontology analysis of protein-coding genes altered in cells by coculture. For each category the top 10 most significantly enriched GO terms (FDR < 0.05) are displayed. BP = Biological Process, CC = Cellular Compartment, MF = Molecular Function. Individual values can be found in Additional file 17. (B) Volcano plot of log2 fold changes by RNA-Seq of mRNA and lncRNA genes (combined) in EVs derived from tumor-exposed cells vs. EVs derived from unexposed cells. (C) Log2 fold change in tumor-exposed cells vs. unexposed cells and in EVs derived from tumor-exposed cells vs. EVs derived from unexposed cells for the most increased (left) and decreased (right) mRNA and lncRNA genes significantly changed in cells. Error bars represent standard error of log2 fold change. Individual values can be found in Additional file 17. (D-E) Depletion of E2F_TARGETS_V1 gene set (D) and G2M_TARGETS gene set (E) in tumor-exposed vs. unexposed cells and enrichment of the same gene set in EVs derived from tumor-exposed vs. unexposed cells. All analyses were performed using 3 EV and 3 cell samples. [file 12915_2022_1277_MOESM14_ESM.pdf]

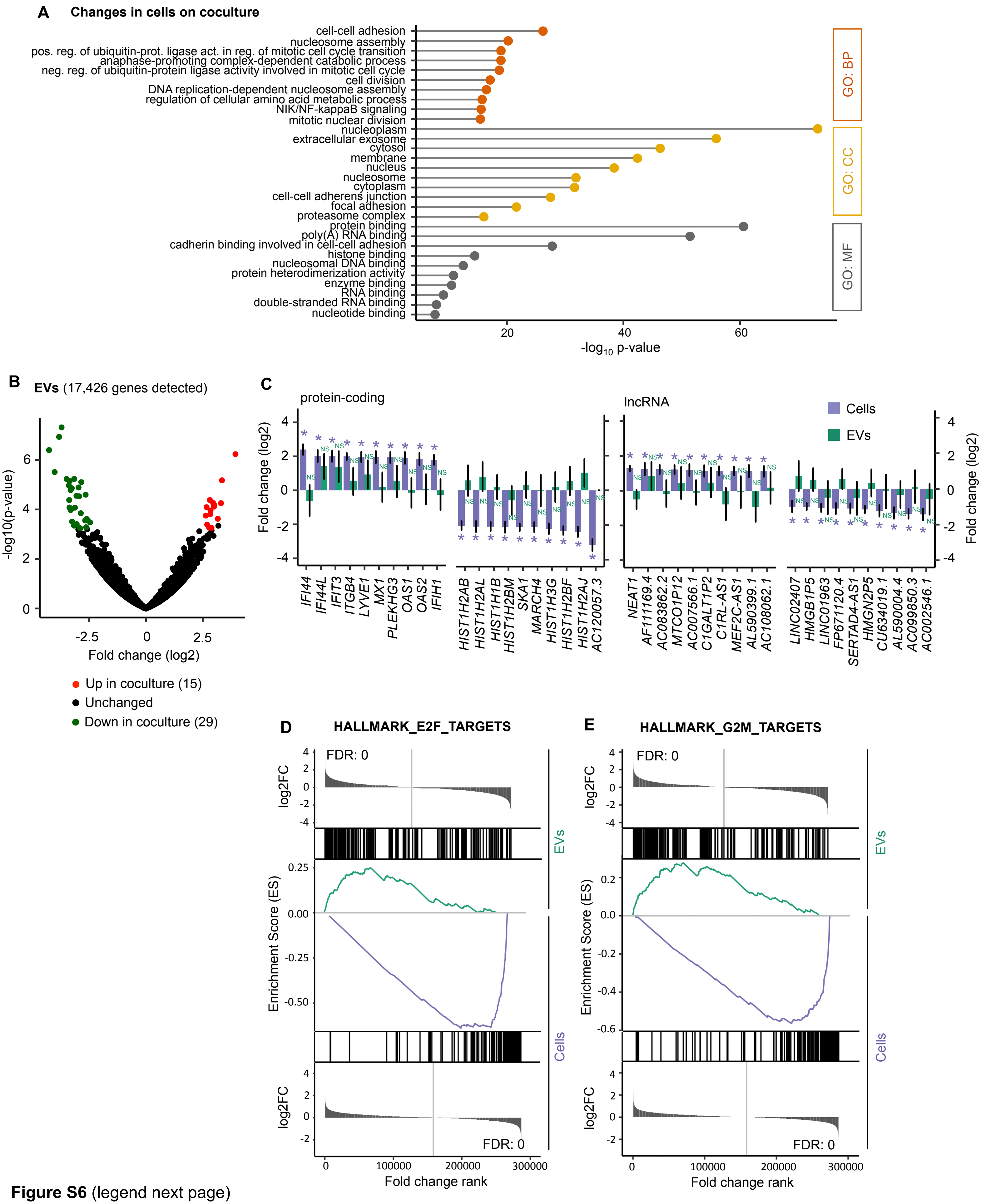

Figure S6 (legend next page)

Figure S6: (A) Gene Ontology analysis of protein-coding genes altered in cells by coculture. For each category the top 10 most significantly enriched GO terms (FDR < 0.05) are displayed. BP = Biological Process, CC = Cellular Compartment, MF = Molecular Function. Individual values can be found in Additional file 17. (B) Volcano plot of log2 fold changes by RNA-Seq of mRNA and lncRNA genes (combined) in EVs derived from tumor-exposed cells vs. EVs derived from unexposed cells. (C) Log2 fold change in tumor-exposed cells vs. unexposed cells and in EVs derived from tumor-exposed cells vs. EVs derived from unexposed cells for the most increased (left) and decreased (right) mRNA and lncRNA genes significantly changed in cells. Error bars represent standard error of log2 fold change. Individual values can be found in Additional file 17. (D-E) Depletion of E2F_TARGETS_V1 gene set (D) and G2M_TARGETS gene set (E) in tumor-exposed vs. unexposed cells and enrichment of the same gene set in EVs derived from tumor-exposed vs. unexposed cells. All analyses were performed using 3 EV and 3 cell samples.
