# Supplementary material for: Sorting and packaging of RNA into extracellular vesicles shape intracellular transcript levels
Source: BMC Biol. 2022 Mar 24;20:72. doi: 10.1186/s12915-022-01277-4 (PMC8944098; doi:10.1186/s12915-022-01277-4)
Supplement: Supplementary file 15 — Additional file 15: Figure S7. (A) Western blot of lysate from cells treated with GW4869 or DMSO and from EVs derived from cells treated with GW4869 or DMSO, using indicated antibodies (left). (B-C) Distributions of RNA-Seq log2 fold changes of (B) protein-coding genes and (C) lncRNA enriched in EVs (green) and not enriched in EVs (purple) in cells treated with GW4869. N = 3. (D) Western blot of lysates from cells treated with Src Inhibitor 1 or DMSO and from EVs derived from cells treated with Src inhibitor 1 or DMSO, using antibodies to SDCBP (exosome marker) or tubulin (control). (E) Western blot of lysates from cells treated with ketoconazole or DMSO and from EVs derived from from cells treated with keotconazole or DMSO, using antibodies to SDCBP (exosome marker) or tubulin (control). (F) Enrichment in EVs vs. cells by RNA-Seq (green scale; n = 3) and indication of GGAG motif, UAG motif or HNRNPA2B1 CLIP peak presence in indicated transcripts. (G-I) Western blots of lysates from cells treated with control siRNA (siCTRL) in comparison with cells treated with (G) siHNRNPA2B1 #1, (H) siHNRNPA2B1 #2 or (I) siHNRNPA1 using indicated antibodies. Uncropped images of blots can be found in Additional file 18. [file 12915_2022_1277_MOESM15_ESM.pdf]

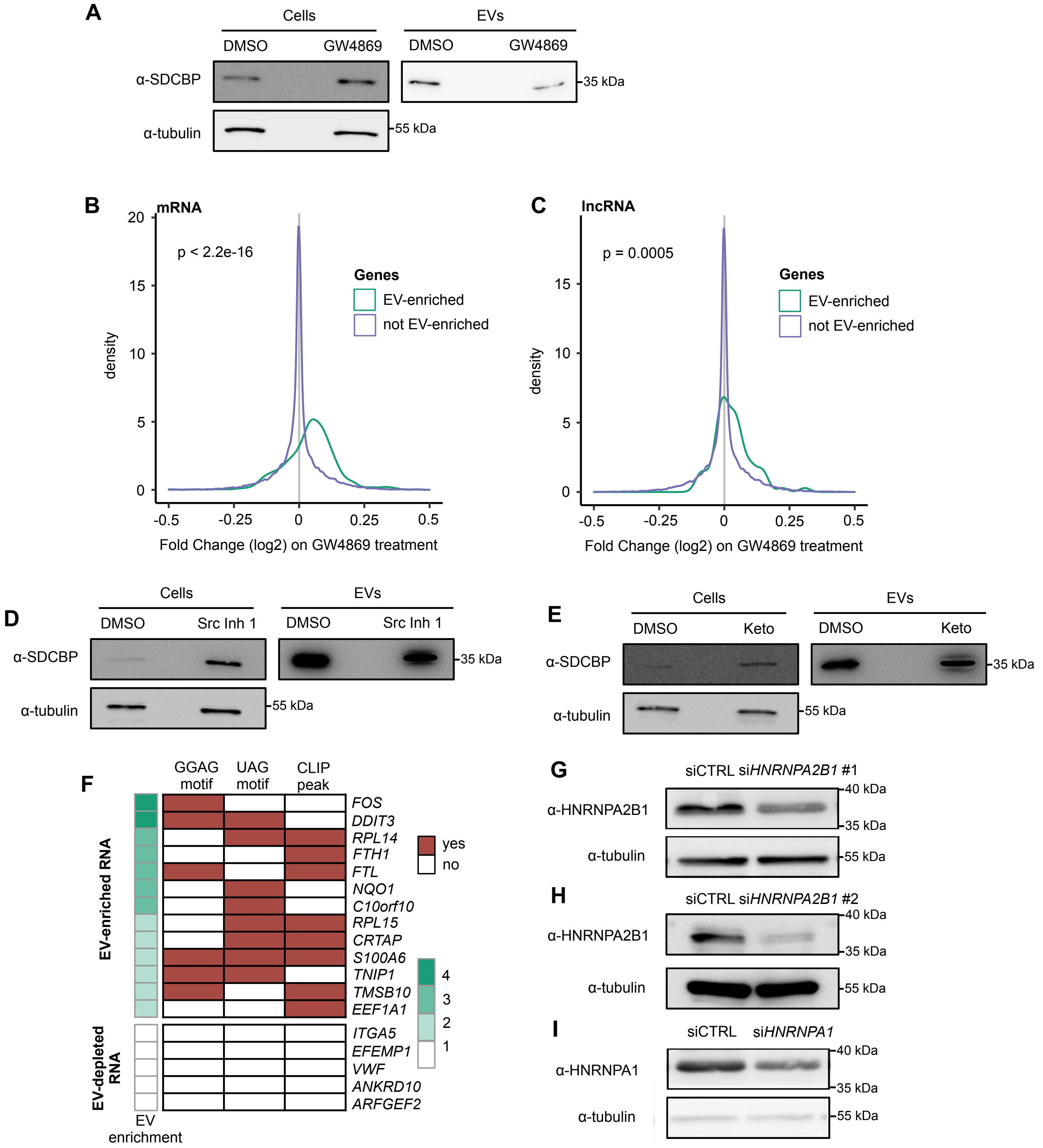

Figure S7: (A) Western blot of lysate from cells treated with GW4869 or DMSO and from EVs derived from cells treated with GW4869 or DMSO, using indicated antibodies (left). (B-C) Distributions of RNA-Seq log2 fold changes of (B) protein-coding genes and (C) lncRNA enriched in EVs (green) and not enriched in EVs (purple) in cells treated with GW4869. N = 3. (D) Western blot of lysates from cells treated with Src Inhibitor 1 or DMSO and from EVs derived from cells treated with Src inhibitor 1 or DMSO, using antibodies to SDCBP (exosome marker) or tubulin (control). (E) Western blot of lysates from cells treated with ketoconazole or DMSO and from EVs derived from from cells treated with keotconazole or DMSO, using antibodies to SDCBP (exosome marker) or tubulin (control). (F) Enrichment in EVs vs. cells by RNA-Seq (green scale; n = 3) and indication of GGAG motif, UAG motif or HNRNPA2B1 CLIP peak presence in indicated transcripts. (G-I) Western blots of lysates from cells treated with control siRNA (siCTRL) in comparison with cells treated with (G) si*HNRNPA2B1* #1, (H) si*HNRNPA2B1* #2 or (I) si*HNRNPA1* using indicated antibodies. Uncropped images of blots can be found in Additional file 18.
