# Supplementary material for: Sorting and packaging of RNA into extracellular vesicles shape intracellular transcript levels
Source: BMC Biol. 2022 Mar 24;20:72. doi: 10.1186/s12915-022-01277-4 (PMC8944098; doi:10.1186/s12915-022-01277-4)

Figure S1A

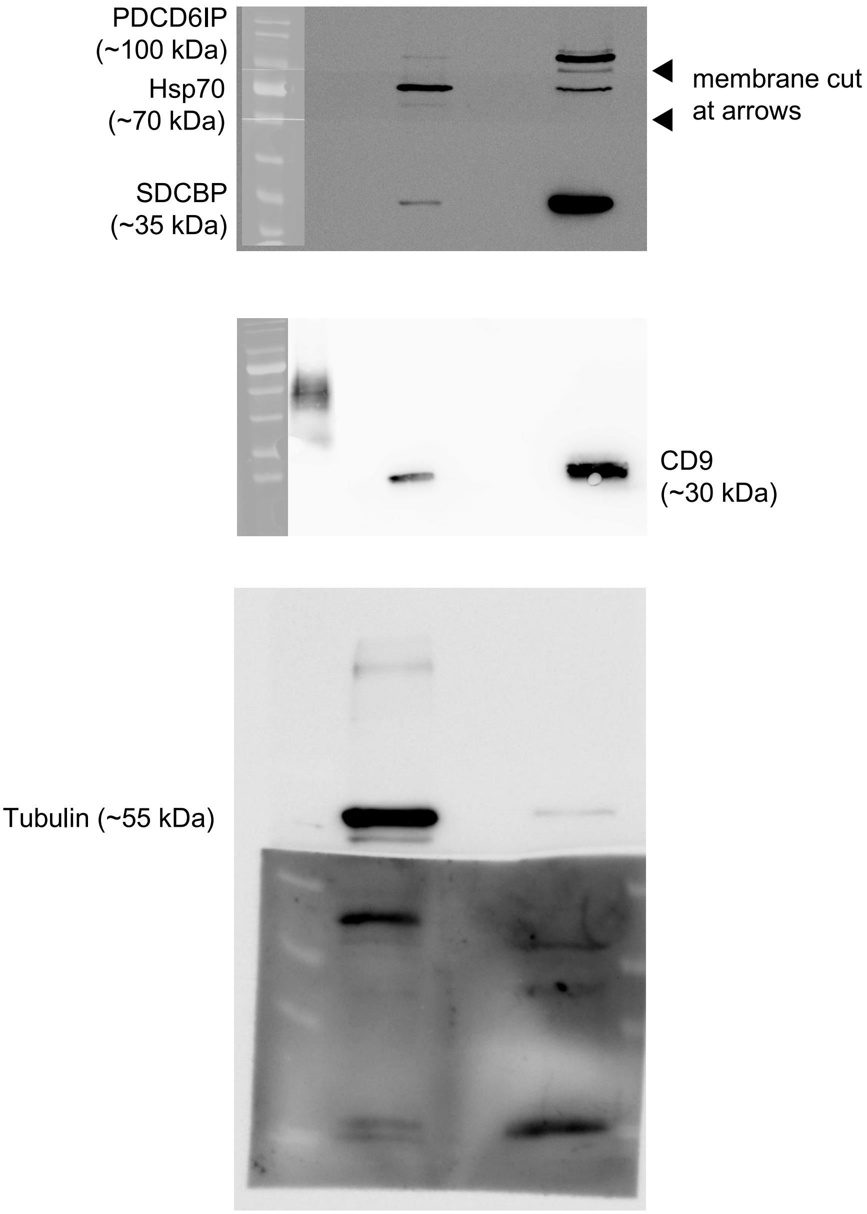
PDCD6IP
(~100 kDa)
Hsp70
(~70 kDa)
SDCBP
(~35 kDa)
membrane cut
at arrows
CD9
(~30 kDa)
Tubulin (~55 kDa)

Figure 2G

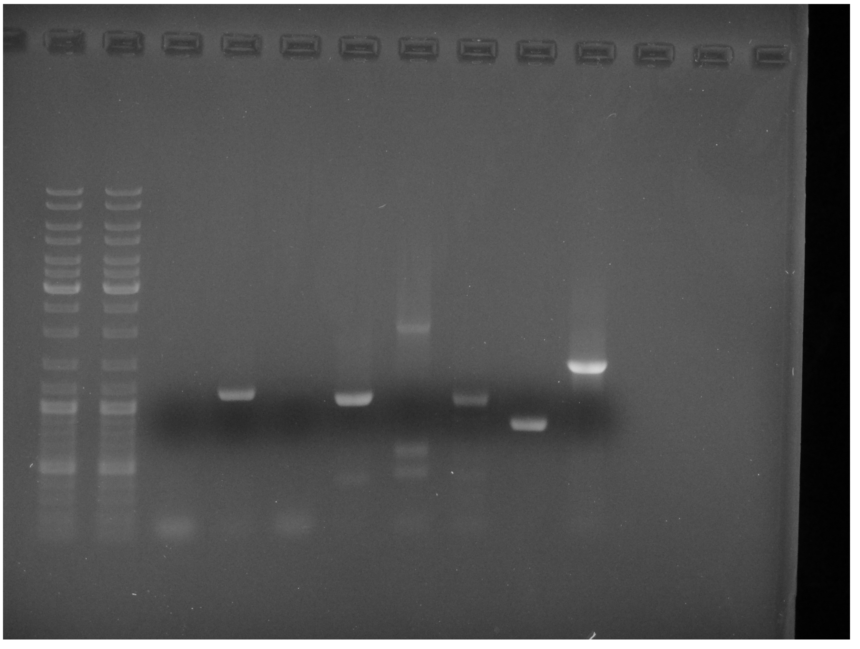

Figure 2H

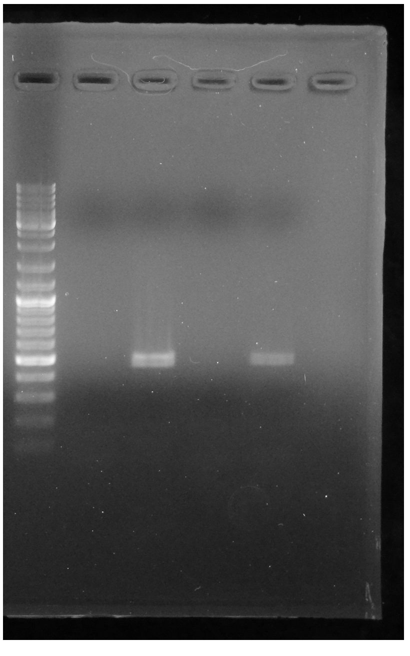

Figure S2C (HNRNPA1)

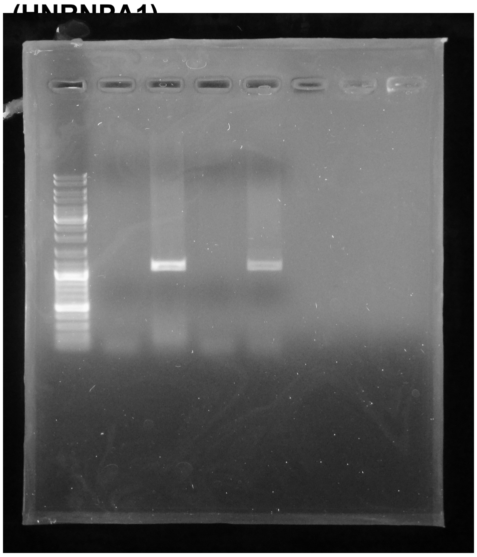

Figure S2D (ANP32B)

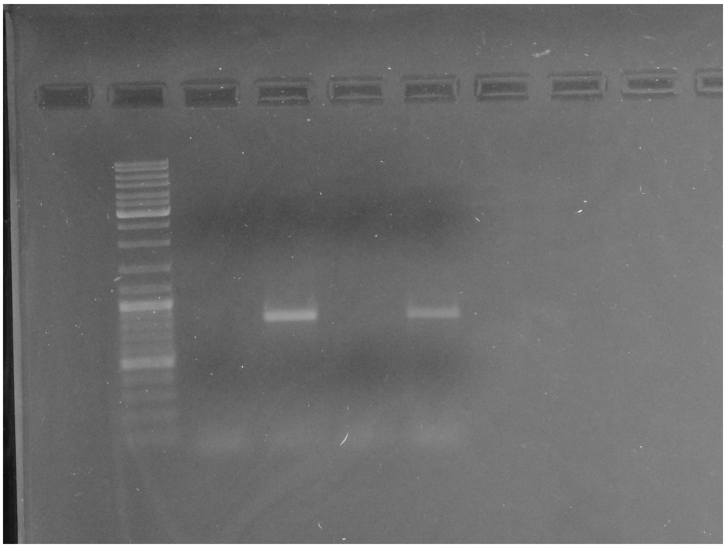

Figure S2E (RPL14)

Figure S2F (RPL41)

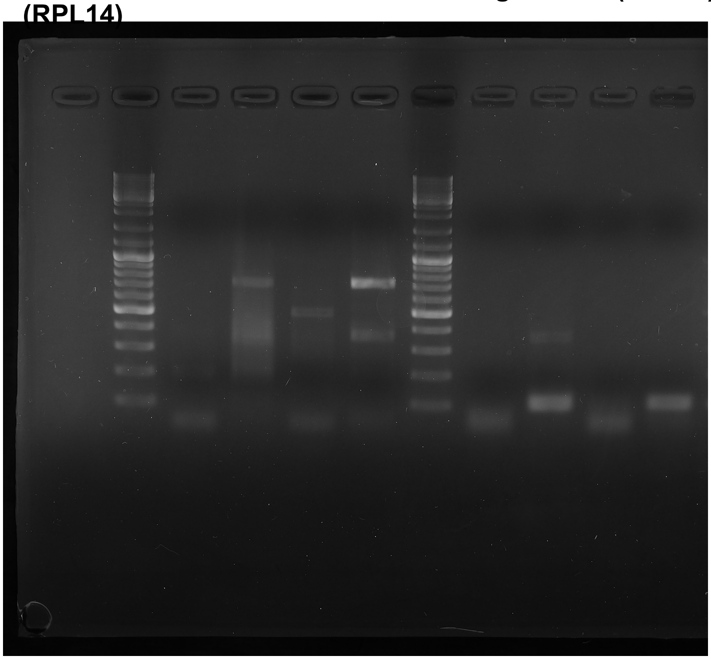

Figure S2G (GAS5)

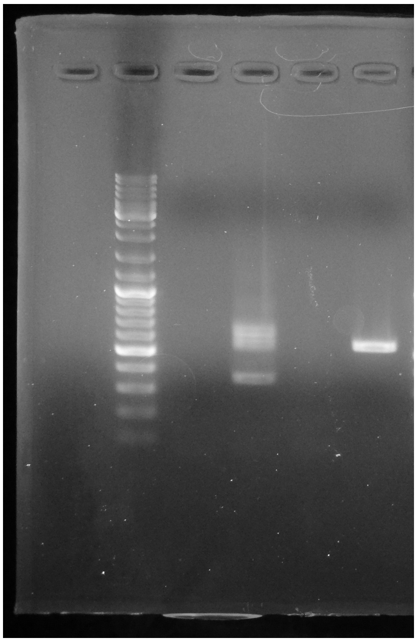

Figure S7A (GW4869)

Tubulin
(~55 kDa)

SDCBP
(~35 kDa)

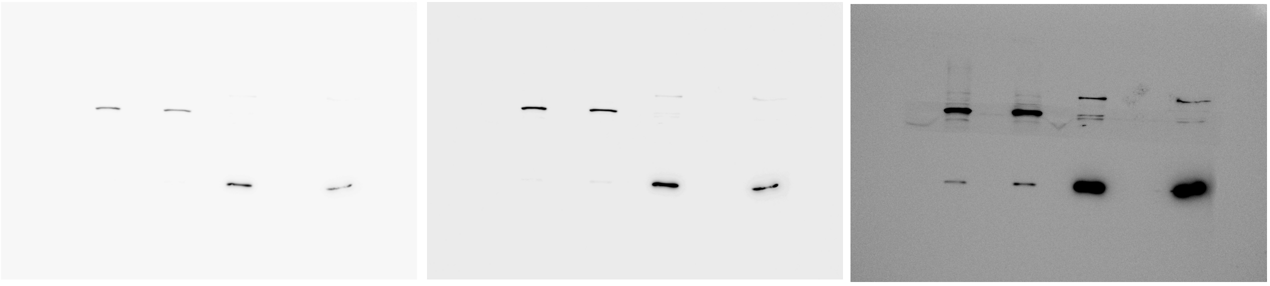

Figure S7D (Src Inhibitor 1)

membrane cut at
arrows; other
antibodies used
in upper sections

SDCBP
(~35 kDa)

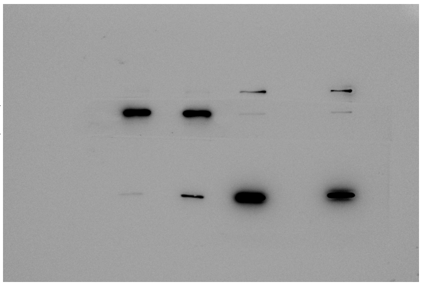

Tubulin (~55 kDa) in multiple
gels/membranes imaged at once

Src Inhibitor 1
treated

ketoconazole
treated

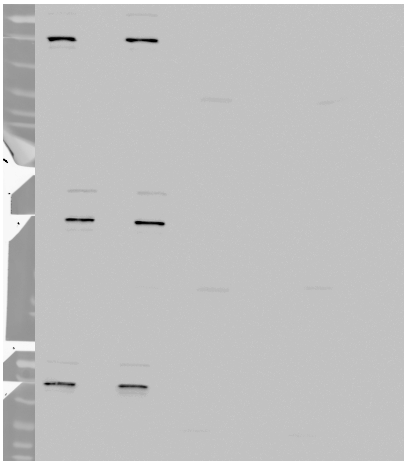

Figure S7E (ketoconazole)

membrane cut at
arrows; other
antibodies used
in upper sections

SDCBP
(~35 kDa)

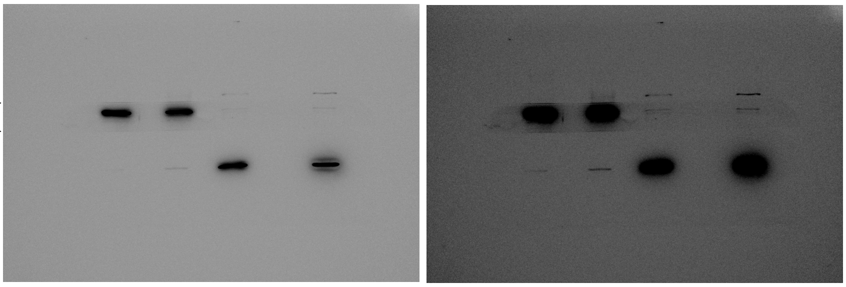

Figure S7G

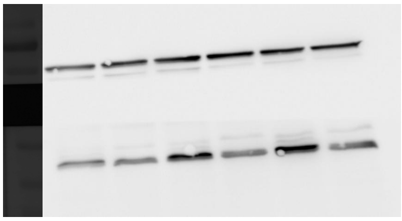

Figure S7H

Tubulin (~55 kDa)
HNRNPA2B1 (~37 kDa)

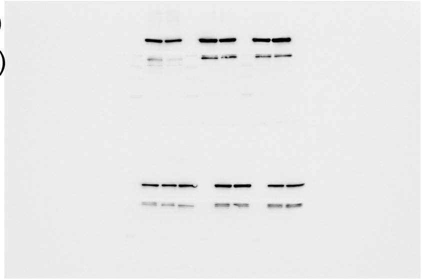

Figure S7I

HNRNPA1
(~37 kDa)

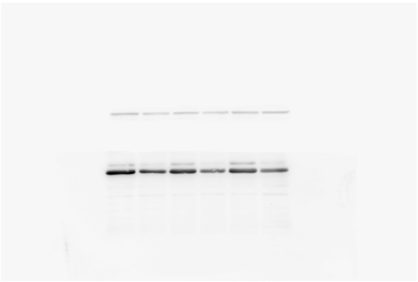

Supplement: Supplementary file 18 — Additional file 18. Uncropped gels and blots. [file 12915_2022_1277_MOESM18_ESM.pdf]
