# Supplementary material for: Sorting and packaging of RNA into extracellular vesicles shape intracellular transcript levels
Source: BMC Biol. 2022 Mar 24;20:72. doi: 10.1186/s12915-022-01277-4 (PMC8944098; doi:10.1186/s12915-022-01277-4)
Supplement: Supplementary file 19 — Table S1. PCR primers [file 12915_2022_1277_MOESM19_ESM.pdf]

Table S1.

PCR primers

Gene	Forward (5' to 3')	Reverse (5' to 3')	Type	Citation
ANKRD10	CAGAGCAGTGTACATTCTTGTCTT	ACTGGCATTTCTCAGGCTTT	qRT-PCR	N/A
ANP32B	ACTTGGATGGCTATGACCGAG	TCCTCACTGACTTCATCGTCG	qRT-PCR	N/A
ARFGEF2	CCTCTGGCAGGTGACTTTTG	GTCACACTTGTACTTATCAGCAGG	qRT-PCR	N/A
CRTAP	ACAAGTTCCTGCAGTTCGCT	TAGAAAGGTGTGAGCAGCGG	qRT-PCR	N/A
DEPP1 (c10orf10)	ACTGGACTGTCCCTGCTCAT	CCGTGGTCTCCCGAATTGTG	qRT-PCR	N/A
DDIT3	TGGAAAGCAGCGCATGAAGGA	AAAGGTGGGTAGTGTGGCCCAA	qRT-PCR	N/A
EEF1A1	TGTCGTCATTGGACACGTAGA	ACGCTCAGCTTTCAGTTTATCC	qRT-PCR	N/A
EFEMP1	ACCCTTCCCACCGTATCCA	TCTGCTCTACAGTTGTGCGTCC	qRT-PCR	[1]
FOS	AGGAGGGAGCTGACTGATACACT	TTTCCTTCTCCTTCAGCAGGTT	qRT-PCR	N/A
FRMD5	GCTGCATTTCTGGCCTTCAC	ACACGCTTCAGGAGTTGGAG	qRT-PCR	N/A
FTH1	GACTCAGAGGCCGCCATCAA	AAGATTCGGCCACCTCGTTG	qRT-PCR	N/A
FTL	CAACCAACCATGAGCTCCCA	AATTCGCGGAAGAAGTGGCT	qRT-PCR	N/A
GPR107	TGGTCGACCTGTTGTGTTGT	CCCATGCTGTCACCTTTTCC	qRT-PCR	N/A
HDAC5	TTGGAGACGTGGAGTACCTTACAG	GACTAGGACCACATCAGGTGAGAAC	qRT-PCR	N/A
HMOX1	TGACCCATGACACCAAGGAC	AGTGTAAGGACCCATCGGAGA	qRT-PCR	N/A
HOOK2	TCGAGGAGCTGCAGCATAAC	CTGCTTGGGTTCCATGGTCT	qRT-PCR	N/A
ITGA5	GTCGGGGGCTTCAACTTAGAC	CCTGGCTGGCTGGTATTAGC	qRT-PCR	[2]
NQO1	GGTTTGGAGTCCCTGCCATT	ACCAGTGGTGATGGAAAGCA	qRT-PCR	N/A
NR4A1	GCTGCAGAATGACTCCACC	ACAGCAGCACTGGGCTTA	qRT-PCR	N/A
RAB13	CGCACTGTGGATATAGAGGGG	CCATGGCTCCACGGTAGTAG	qRT-PCR	N/A
RN7SL1	ATCGGGTGTCCGCACTAAG	CACCCCTCCTTAGGCAACCT	qRT-PCR	N/A
RPL4	AAGATCCATCGCAGAGTCCT	TTATCCACCCGGAGCTTGTG	qRT-PCR	N/A
RPL14	TCATGCCGGAAAATTGGTCG	CATGGCCTGTCTCCTCACTT	qRT-PCR	N/A
RPL15	CTGGGTTGGTGAAGATTCCA	GTGGACTGGTTTGGTGATCC	qRT-PCR	[3]
RPL26	CGGAAGCAGCGTGTAGTTCT	TCGGTCGGAAGTCACAAAGG	qRT-PCR	N/A
RPL38	TTTCGTCCTTTTCCCCGGTT	CAATTTTCCGAGGCATGGCG	qRT-PCR	N/A
RPL41	GCCTTTCTCTCGGCCTTAGC	TTCTTCTTTTGCGCTTCAGCC	qRT-PCR	N/A
S100A6	GGGAGGGTGACAAGCACAC	AGCTTCGAGCCAATGGTGAG	qRT-PCR	[4]
TCIRG1	CAGCTCTTTCTGCCCACAG	CTGCAGGAAGGTGAAGGTCT	qRT-PCR	[5]
THBS1	CGGTCCAGACACGGACCTGC	GGCTTTGGTCTCCCGCGCTT	qRT-PCR	[6]
TMSB10	TGGCAGACAAACCAGACATGG	CGAAGAGGACGGGGGTAGG	qRT-PCR	N/A
TNIP1	AATACACCTGGCGTCTACCC	ATGAAGGTGGAGCCAAATGACA	qRT-PCR	N/A
TPT1	GATCGCGGACGGGTTGT	TTCAGCGGAGGCATTTCC	qRT-PCR	[7]

VWF	AGTGAGCCTCTCCGTGTATC	TCACCGGACAGCTTGTAGTA	qRT-PCR	[8]
ANP32B	AGGGGAACATGGACATGAAG	AATCCATGAGCAGTCCAACC	PCR	N/A
EEF1A1	CCAGAACACAGGTGTCGTG	TCATTTAGCCTTCTGAGCTTTCTG	PCR	N/A
GAS5	CGACTCCTGTGAGGTATGGT	TTCTTGTGCCATGAGACTCC	PCR	N/A
hnRNPA1	CCCTGCCGTCATGTCTAAGT	GTGCTTGGCTGAGTTCACAA	PCR	N/A
RPL14	CTAACGCCGCCAACATGG	GCCACTTATGCTTTCTTGCCAG	PCR	N/A
RPL41	CCTCTGCGCCATGAGAGC	CAAGCTAGCGGTTTACTTGGACC	PCR	N/A
SNHG5	GCGGGTGGTAGGAACAAT	ATGCTCCCCATGTTATTTCA	PCR	N/A

References

1. Chen J, Wei D, Zhao Y, Liu X, Zhang J: **Overexpression of EFEMP1 correlates with tumor progression and poor prognosis in human ovarian carcinoma.** *PLoS One* 2013, **8:**e78783.
2. Wong AW, Paulson QX, Hong J, Stubbins RE, Poh K, Schrader E, Nunez NP: **Alcohol promotes breast cancer cell invasion by regulating the Nm23-ITGA5 pathway.** *J Exp Clin Cancer Res* 2011, **30:**75.
3. Yan TT, Fu XL, Li J, Bian YN, Liu DJ, Hua R, Ren LL, Li CT, Sun YW, Chen HY, et al: **Downregulation of RPL15 may predict poor survival and associate with tumor progression in pancreatic ductal adenocarcinoma.** *Oncotarget* 2015, **6:**37028-37042.
4. Feng S, Zhou Q, Yang B, Li Q, Liu A, Zhao Y, Qiu C, Ge J, Zhai H: **The effect of S100A6 on nuclear translocation of CacyBP/SIP in colon cancer cells.** *PLoS One* 2018, **13:**e0192208.
5. Thudium CS, Moscatelli I, Löfvall H, Kertész Z, Montano C, Bjurström CF, Karsdal MA, Schulz A, Richter J, Henriksen K: **Regulation and Function of Lentiviral Vector-Mediated TCIRG1 Expression in Osteoclasts from Patients with Infantile Malignant Osteopetrosis: Implications for Gene Therapy.** *Calcif Tissue Int* 2016, **99:**638-648.
6. Bonazzi VF, Nancarrow DJ, Stark MS, Moser RJ, Boyle GM, Aoude LG, Schmidt C, Hayward NK: **Cross-platform array screening identifies COL1A2, THBS1, TNFRSF10D and UCHL1 as genes frequently silenced by methylation in melanoma.** *PLoS One* 2011, **6:**e26121.
7. Andersen CL, Jensen JL, Ørntoft TF: **Normalization of real-time quantitative reverse transcription-PCR data: a model-based variance estimation approach to identify genes suited for normalization, applied to bladder and colon cancer data sets.** *Cancer Res* 2004, **64:**5245-5250.
8. Maleszewska M, Moonen JR, Huijkman N, van de Sluis B, Krenning G, Harmsen MC: **IL-1β and TGFβ2 synergistically induce endothelial to mesenchymal transition in an NFκB-dependent manner.** *Immunobiology* 2013, **218:**443-454.
